# Hybrid Plasmonic Nanostructures for Enhanced Single-Molecule Detection Sensitivity

**DOI:** 10.1021/acsnano.3c00576

**Published:** 2023-04-03

**Authors:** Ediz Kaan Herkert, Domenica Romina Bermeo Alvaro, Martina Recchia, Wolfgang Langbein, Paola Borri, Maria F. Garcia-Parajo

**Affiliations:** † ICFO-Institut de Ciencies Fotoniques, The Barcelona Institute of Science and Technology, 08860 Castelldefels (Barcelona), Spain; ‡ School of Biosciences, Cardiff University, Museum Avenue, CF10 3AX Cardiff, United Kingdom; § School of Physics and Astronomy, Cardiff University, The Parade, Cardiff CF24 3AA, United Kingdom; ∥ ICREA, Pg. Lluís Companys 23, 08010 Barcelona, Spain

**Keywords:** optical nanoantennas, plasmonic biosensing, electron beam lithography, hybrid materials, plasmonics

## Abstract

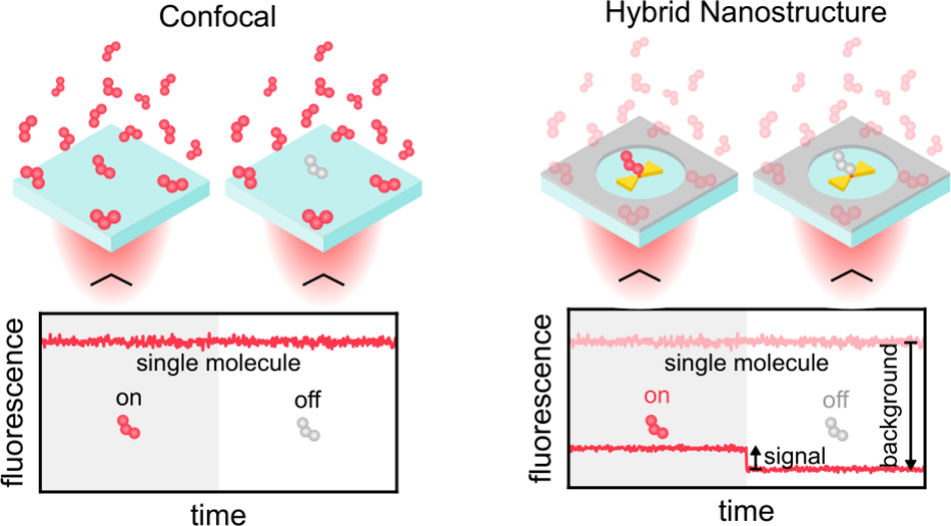

Biosensing applications based on fluorescence detection often require single-molecule sensitivity in the presence of strong background signals. Plasmonic nanoantennas are particularly suitable for these tasks, as they can confine and enhance light in volumes far below the diffraction limit. The recently introduced antenna-in-box (AiB) platforms achieved high single-molecule detection sensitivity at high fluorophore concentrations by placing gold nanoantennas in a gold aperture. However, hybrid AiB platforms with alternative aperture materials such as aluminum promise superior performance by providing better background screening. Here, we report on the fabrication and optical characterization of hybrid AiBs made of gold and aluminum for enhanced single-molecule detection sensitivity. We computationally optimize the optical properties of AiBs by controlling their geometry and materials and find that hybrid nanostructures not only improve signal-to-background ratios but also provide additional excitation intensity and fluorescence enhancements. We further establish a two-step electron beam lithography process to fabricate hybrid material AiB arrays with high reproducibility and experimentally validate the higher excitation and emission enhancements of the hybrid nanostructures as compared to their gold counterpart. We foresee that biosensors based on hybrid AiBs will provide improved sensitivity beyond the capabilities of current nanophotonic sensors for a plethora of biosensing applications ranging from multicolor fluorescence detection to label-free vibrational spectroscopy.

Monitoring dynamic biological processes on ever-smaller scales allows researchers to form a more detailed understanding of how physiological and pathological processes take place. These processes are often governed by the dynamic interaction between individual biomolecules, and thus methods enabling the observation of single molecules at relevant physiological concentrations and spatiotemporal scales are highly desirable. Continuous technological developments have made it possible to access the single-molecule regime by noninvasive optical means.^1−3^ The great majority of these optical approaches achieve the required sensitivity and specificity by exploiting the fluorescence contrast provided by organic dyes, autofluorescent proteins, or quantum dots exclusively binding to the biomolecule of interest. However, conventional fluorescence methods are performed in the far field and thus are subject to the diffraction limit. As illustrated in Figure 1a, this effectively limits the concentration of the target molecule to the submicromolar range,^4^ which is generally below physiologically relevant concentrations.

**Figure 1:**
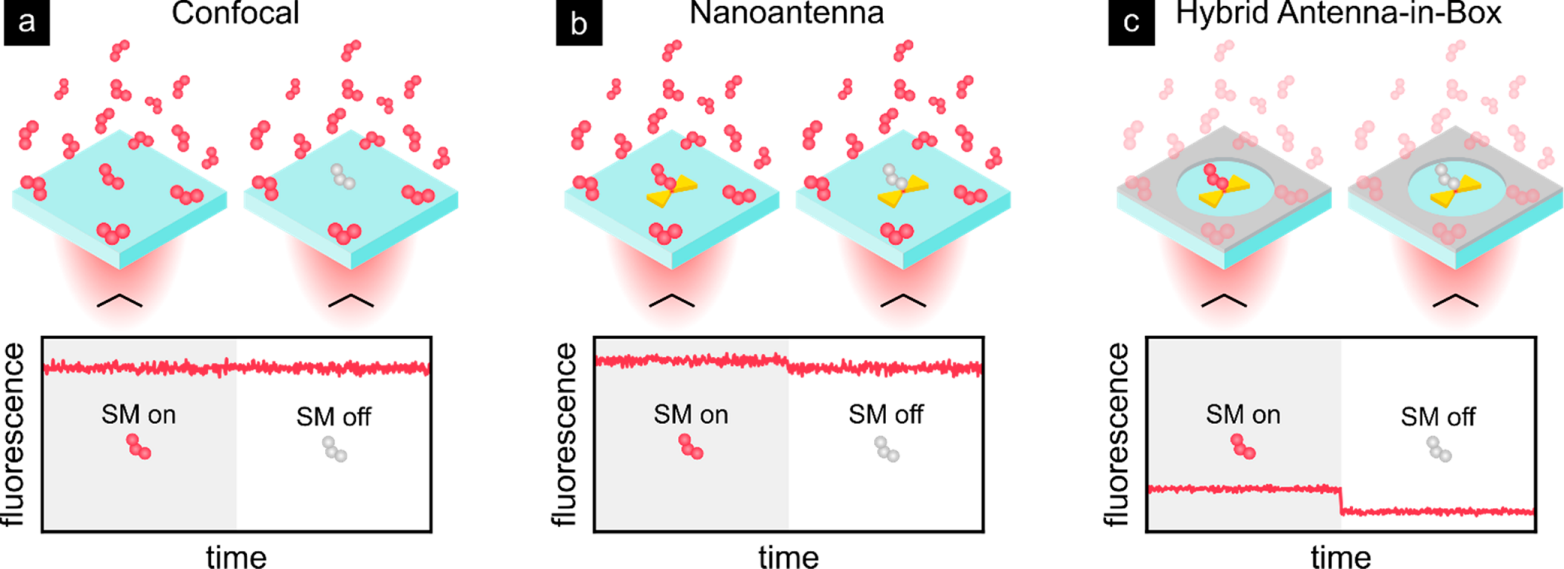
Sketch of the increased single-molecule (SM) sensitivity at high concentrations by different types of nanostructures. (a) In the absence of nanostructures the diffraction-limited confocal beam not only excites the target molecule but also generates a significant fluorescence background from the surrounding excited molecules, prohibiting the detection of single molecules. (b) Nanoantennas enhance the light in subdiffraction volumes increasing the signal from the target molecule without reducing the fluorescence background. (c) Antenna-in-box nanostructures simultaneously reduce the fluorescence background by attenuating the excitation beam and increase the signal enhancement as compared to conventional nanoantennas. Employing hybrid material platforms can boost both effects.

Nanophotonic biosensors based on metallic nanostructures have gained popularity in recent years, as they are capable of confining and enhancing light in subdiffraction volumes through the excitation of plasmonic resonances.^5−24^ The confined high-intensity observation volumes provided by the nanostructures enable pushing the concentration limit toward the physiologically relevant regime and thus observing individual target molecules in their native environment. Metallic nanostructures can manipulate not only the excitation of a molecule by confining and enhancing the excitation field but also its emission by modifying the quantum yield.^25,26^ The latter originates from two competing processes introduced by the nanostructures. On the one hand, the metal introduces additional loss channels due to absorption including Ohmic losses, which will reduce the effective quantum yield of the fluorophore. On the other hand, the radiative decay rate can be increased by the Purcell effect owing to an increased local density of optical states (LDOS) close to the nanostructures at the emission wavelength, increasing the quantum yield particularly for emitters with low intrinsic quantum yield. Therefore, in typical experimental scenarios, nanostructures increase the quantum yield of weakly fluorescent emitters while decreasing it for fluorescent emitters with a high intrinsic quantum yield.^9,27,28^ Notably, the increased radiative decay rates allow for larger saturated emission rates and thus higher photon detection rates.

For these reasons, metallic nanostructures are particularly useful in fluorescence-based applications that require the detection of individual weakly fluorescent molecules at micro- to millimolar concentrations (intermolecule distances of 10–100 nm). Nonetheless, at such high concentrations, simple nanostructure designs such as rod or bowtie nanoantennas (BNAs) suffer from a strong fluorescence background that is generated by the large confocal excitation volume surrounding the small volume enhanced by the nanostructure. This issue is illustrated in Figure 1b and can be partially mitigated by employing statistical methods,^18^ using total internal reflection fluorescence (TIRF) illumination,^18,29^ or probing specimens with nanometric axial extent such as lipid bilayers.^11^ Inverse nanoantenna designs provide an alternative path with a significantly reduced fluorescence background at the cost of decreased intensity enhancement. These designs are also known as zero-mode waveguides (ZMW)^5,21^ or nanoapertures^10^ and evanescently confine the excitation light axially and laterally in relatively small observation volumes. This makes them more suitable for studies with spatially extended specimens that would otherwise generate a strong fluorescence background.^8,10^

In 2013 Punj et al.^28^ introduced antenna-in-box (AiB) platforms that combine the strengths of nanoantennas with the strong background reduction of their inverse counterparts. For this, they fabricated gold (Au) dimer disk nanoantennas embedded within a rectangular gold nanoaperture and showed strong background screening and enhanced fluorescence, leading to increased single-molecule detection sensitivity at micromolar sample concentrations (Figure 1c). Due to their capabilities various authors adapted this biosensor platform design for their work. Flauraud et al.^13^ presented an alternative approach to fabricate large arrays of planarized AiBs using electron beam lithography (EBL) and template stripping. They demonstrated that even higher fluorescence enhancement factors and smaller observation volumes are possible as compared to the initial focused-ion-beam (FIB)-based approach. Importantly, EBL-based AiBs enabled meaningful biological membrane studies, as the large number of planar AiBs allowed acquiring statistics over many individual antennas while minimizing potential artifacts on the molecule diffusion induced by nonplanar nanostructures. The suitability of this second generation of AiB platforms for biosensing applications was initially demonstrated by Winkler et al.^14^ on model lipid membranes and then applied to living cell membrane studies.^12,15^

Further improvements on the signal-to-background ratio (SBR) and signal enhancement could be obtained by optimizing the geometry of the AiBs and, importantly, by introducing material combinations that preserve the advantages of gold in terms of signal enhancement, while improving the background suppression by using materials with lower transmission in the visible regime such as aluminum (Al). Besides their superior performance, such optimized hybrid material devices could become particularly relevant in multicolor fluorescence applications which are so far limited to the red and near-infrared spectral range in gold-based nanostructures.^30^ Moreover, surface-enhanced vibrational spectroscopy could largely benefit from the high intensity enhancement provided by the AiB platforms due to the very low Raman scattering cross sections.^23,31−35^

Here, we demonstrate the spectral optimization of AiB platforms consisting of a BNA located in a circular nanoaperture for the detection of individual fluorescent molecules toward high signal enhancement factors and SBRs. Using finite-difference time-domain (FDTD) simulations, we show that the nanoaperture diameter controls the excitation intensity enhancement, establishing an optimal trade-off between signal enhancement and SBR. Moreover, we find that hybrid Au-Al-AiBs consisting of a Au-BNA inside an Al-aperture outperform isolated Au-BNAs and Au-Au-AiBs in three crucial figures of merit: (i) excitation intensity enhancement, (ii) fluorescence enhancement, and (iii) SBR enhancement. We further present a two-step EBL overlay process to fabricate large arrays of Au-Au-AiBs and hybrid Au-Al-AiBs and experimentally assess their performance through transmission cross-section spectra and fluorescence enhancement and lifetime measurements.

## Results and Discussion

**Figure 2:**
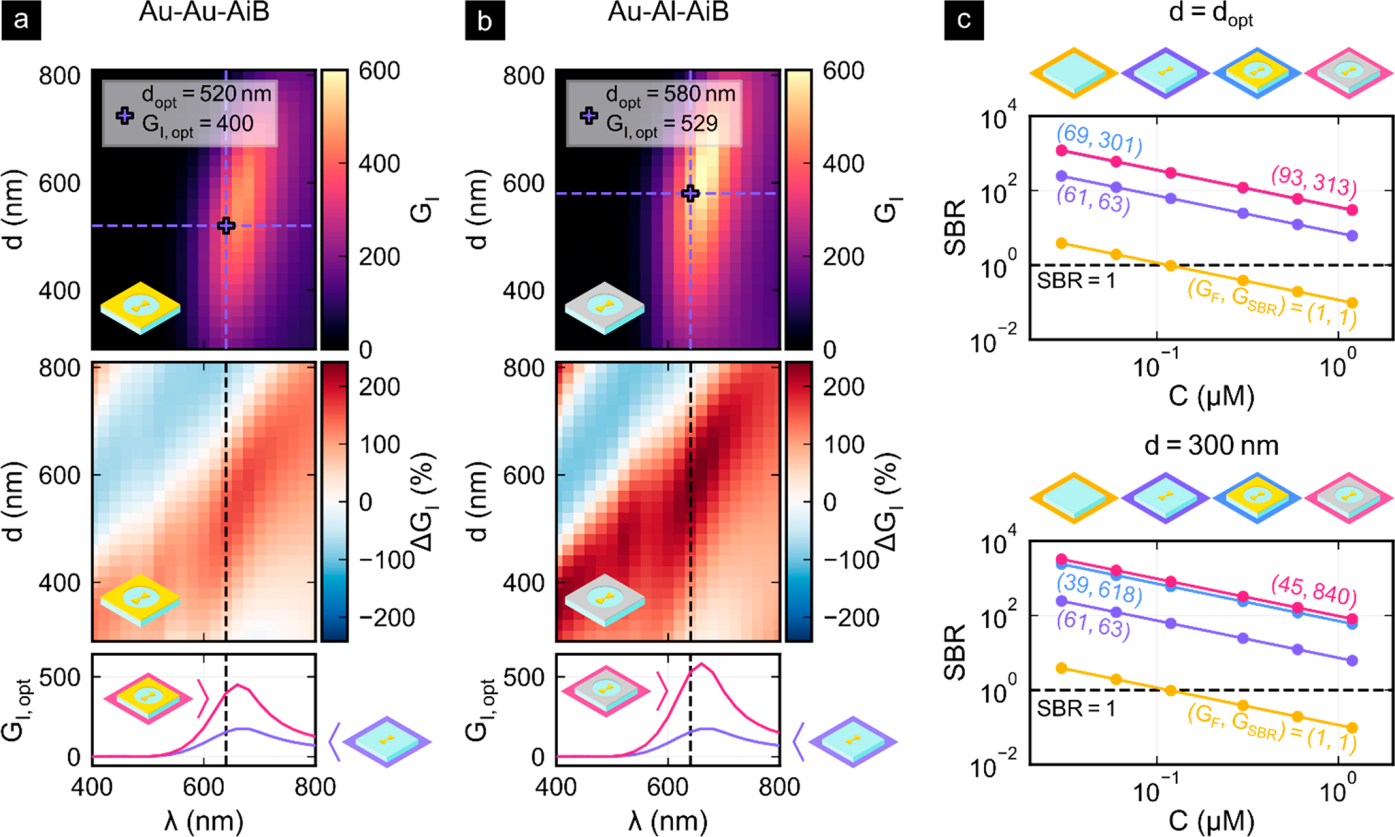
FDTD simulations of (a, b) the spectral excitation intensity enhancements *G*_I_ and (c) fluorescence signal-to-background ratio (SBR). The top rows of (a) and (b) show the dependence of the excitation intensity enhancement on the aperture diameter *d* and the optimal diameter (*d*_opt_) of maximal enhancement at λ_exc_ = 640 nm. The relative enhancements Δ*G*_I_ in the central rows indicate the regimes in which the AiB apertures provide additional gains or losses as compared to isolated BNAs. In the bottom rows, the spectral excitation intensity enhancement for the optimized AiBs is compared to the isolated Au-BNA. The results are shown for the Au-Au-AiB (a) and Au-Al-AiB (b) designs. (c) Signal-to-background ratios at different dye concentrations for the confocal reference (yellow), the Au-BNA (purple), the Au-Au-AiB (blue), and the Au-Al-AiB (red). The top row shows the results when the aperture diameter is *d* = *d*_opt_, and the bottom row corresponds to *d* = 300 nm. *G*_F_ and *G*_SBR_ are displayed in parentheses and indicate the fluorescence and signal-to-background gain, respectively, of the corresponding configuration as compared to the confocal reference. The target dye is located in the hot-spot center and aligned with the excitation polarization for maximum enhancement and emits at λ_emi_ = 676 nm. Due to the similar SBR, the blue line is hidden behind the red line in the upper plot of (c).

The nanostructures developed in this work differ from the original AiB design in the way that they consist of a BNA in a circular aperture instead of disk dimers in a rectangular aperture. We chose this design since BNAs are known to provide high enhancement factors in their gap region,^22,33^ and in addition, to simplify the computational optimization by reducing the number of geometrical parameters of the nanoaperture. Moreover, we included studies of single material Au-Au-AiBs and hybrid material Au-Al-AiBs to assess and compare their performance in terms of excitation intensity enhancement *G*_I_, fluorescence enhancement *G*_F_, and SBR enhancement *G*_SBR_. The numerical optimization of the AiBs shown in Figure 2 was carried out using commercial FDTD software (Lumerical) to maximize the excitation intensity enhancement at the excitation wavelength of λ_exc_ = 640 nm for the Alexa Fluor 647 dye. The Au-BNAs have a length of *l* = 65 nm, a height of *h* = 50 nm, an apex angle of α = 90°, and a gap size of *g* = 20 nm. The simulations shown in Figure S1 of the Supporting Information demonstrate that Au-Au-AiBs and Au-Al-AiBs with this Au-BNA length provide a maximum excitation intensity enhancement at the desired excitation wavelength. An edge and corner curvature radius of *r*_c_ = 20 nm was applied to approximate the fabrication resolution. To accurately reflect the experimental conditions, the nanostructures are on top of BK7 glass and are coated with a 70 nm thick PMMA layer. The gold nanostructures are additionally on top of a 2 nm chromium adhesion layer. A plane wave linearly polarized along the BNA axis was injected from the bottom for excitation. Based on this layout the excitation intensity enhancement
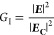
1was computed in Figure 2 for different diameters of the (a) gold and (b) aluminum nanoaperture. Here, ***E*** is the electrical field vector in the center of the BNA gap, and ***E***_**C**_ is the electrical field vector at the same point of the confocal reference simulation without nanostructures. The simulations depicted in the top row of Figure 2a,b clearly show that the nanoaperture diameter affects the excitation intensity enhancement. Importantly, hybrid Au-Al-AiBs provide higher maximum enhancement factors as compared to the Au-Au-AiBs at the optimal diameters *d*_opt_ that maximize the respective excitation intensity enhancement at λ_exc_ = 640 nm. The role of the aperture becomes particularly evident when looking at the relative excitation intensity enhancement
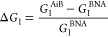
2in the central row of Figure 2a,b that shows the excitation intensity enhancement of the AiBs *G*_I_^AiB^ relative to the BNA excitation intensity enhancement *G*_I_^BNA^. We find that the aperture can both increase or decrease the excitation intensity enhancement in the BNA gap, which can be understood as a coupling of the excitation of the nanoaperture cavity resonances to the BNA plasmon resonance. This is underlined by the similar dependence on the scaled diameter *d*/λ of the relative AiB excitation intensity enhancement Δ*G*_I_(λ,*d*) shown in the central row of Figure 2a,b and the aperture excitation intensity enhancement *G*_I_(λ, *d*) shown in Figure S2 of the Supporting Information and associated text. The bottom row of Figure 2a,b depicts the spectral excitation intensity enhancement of the optimized AiBs compared to that of the isolated BNA. Interestingly, these AiBs show an increased resonance amplitude of Δ*G*_I_ = 1.6 for Au-Au-AiB and Δ*G*_I_ = 2.4 for Au-Al-AiB without a relevant resonance wavelength shift relative to the Au-BNA resonance. These results thus show that the apertures not only provide background screening but can also yield additional excitation intensity enhancement by controlling the aperture diameter.

Figure 2c shows the simulated signal-to-background ratios
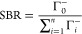
3at different dye concentrations *C*. Here, Γ_*i*_^–^ denotes the fluorescence emission rate of a dipole into the lower hemisphere (epi-detection), with *i* = 0 being the target fluorophore in the BNA gap center with its transition dipole aligned with the BNA axis, and with *i* = 1, ..., *n* being the *n* = 40 background fluorophores randomly distributed and aligned in a 70 nm thick PMMA layer. The nonsaturated fluorescence emission rate
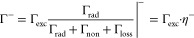
4was computed through the excitation rate Γ_exc_ at the excitation wavelength λ_exc_ = 640 nm and the radiative, nonradiative, and metal loss rates Γ_rad_, Γ_non_, and Γ_loss_, respectively, at the center of the experimental detection window λ_emi_ = 676 nm. η^–^ is the quantum yield of the fluorophore and corresponds to the probability of a photon being emitted in the epi-direction after the absorption of an excitation photon. We chose Γ_non_ = 0 so that the intrinsic quantum yield of the fluorophore is 1 and computed the remaining decay rates as described in Section 3 of the Supporting Information. From these quantities, we derived the fluorescence enhancement
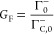
5and the signal-to-background enhancement
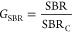
6relative to the confocal reference simulations. Here, Γ_0_^–^ is the emission rate of the fluorophore in the BNA gap center, Γ_C,0_^–^ of the fluorophore in the confocal reference simulation, SBR the signal-to-background ratio of the three types of nanostructures, and SBR_C_ of the confocal reference.

Using the aperture diameters *d*_opt_ of optimal excitation intensity enhancement, the SBRs of both AiB designs are about 5× that of the isolated BNA. Both designs have a similar SBR due to the compensation of the 1.3× higher fluorescence enhancement of the hybrid Au-Al-AiB by the background reduction of the smaller nanoaperture diameter of the Au-Au-AiB. The lower graph of Figure 2c demonstrates that the regime of optimal excitation intensity enhancement does not necessarily coincide with the regime of highest SBR. Especially for applications that suffer from strong background signals such as fluorescence detection, it can be beneficial to trade in signal enhancement for increased background reduction. Because of that, the SBR increases considerably when reducing the nanoaperture diameter to 300 nm despite the reduced fluorescence enhancement. In this case, the SBR enhancement is 9.7 for Au-Au-AiB and 13 for the hybrid Au-Al-AiB as compared to Au-BNA. This means that for hybrid Au-Al-AiB platforms an SBR above unity can be reached at more than 1 order of magnitude higher concentrations as compared to the isolated Au-BNA. We note that the SBR enhancement of the AiB platforms is higher when the background is generated from a specimen with a larger *z*-extent than the 70 nm PMMA layer employed here, as long as it stays below the confocal axial resolution of around 500 nm for high NA objectives. The concentration independence of *G*_SBR_ is explained in Section 3 of the Supporting Information.

As displayed in Table 1, the AiB platforms allow choosing an optimal trade-off between signal enhancement and background reduction. Especially for label-free approaches such as surface-enhanced Raman spectroscopy (SERS),^36^ surface-enhanced coherent anti-Stokes Raman spectroscopy (SECARS),^34^ or surface-enhanced infrared absorption (SEIRA) spectroscopy,^37^ in which high signal enhancement factors are of utmost importance, the ideal trade-off might be closer to the diameter of highest signal enhancement, making AiB designs promising for a wide range of sensing approaches.

**Table 1:** Simulated Figures of Merit for the Different Optimized Nanostructure Designs

		Au-Au-AiB		Au-Al-AiB	
	Au-BNA	*d*_opt_	300 nm	*d*_opt_	300 nm
*G*_I_	155	400	191	529	218
*G*_F_	61	69	39	93	45
*G*_SBR_	63	301	618	313	840

**Figure 3:**
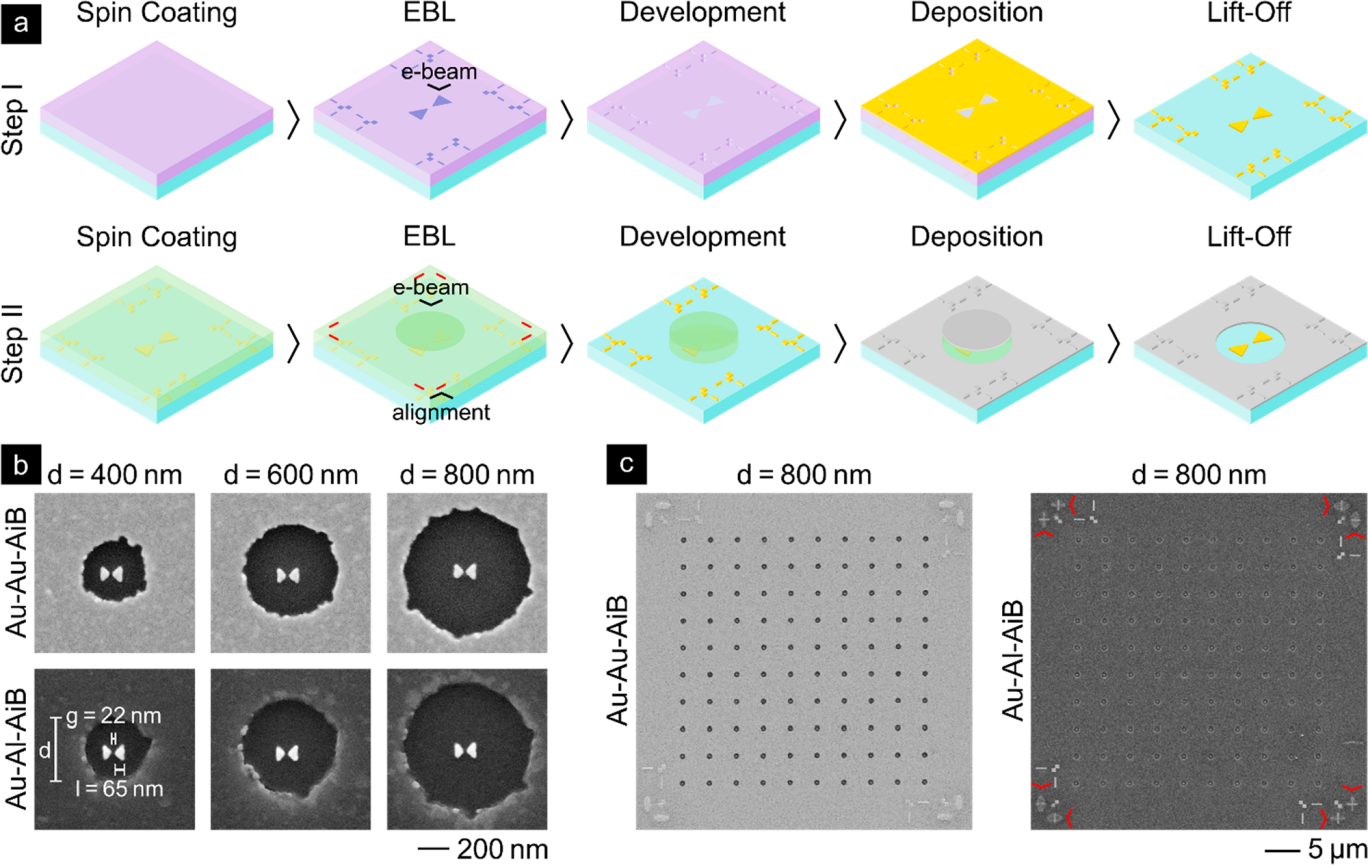
Schematic of the EBL-based fabrication process (a) and SEM images of the Au-Au-AiB and hybrid Au-Al-AiB platforms (b, c). (a) The fabrication process consists of an automated two-step overlay EBL process with a positive- and negative-tone resist. In both steps, the resists are spin-coated on a glass coverslip, exposed in the EBL step, and developed before the metal deposition and lift-off. The only conceptual difference in step II is the use of a negative-tone resist and the automated overlay alignment using the markers fabricated in step I. (b) SEM images of individual Au-Au-AiB and hybrid Au-Al-AiBs for different aperture diameters *d*. The measured BNA length *l* and gap distance *g* are shown on the bottom left of (b). (c) SEM images of the 10 × 10 AiB arrays. The red arrows on the right image of (c) indicate the scanning marks from the alignment process. The SEM images are equally contrast adjusted.

Earlier AiB platforms were fabricated by means of FIB milling^28^ or EBL.^13^ While Flauraud et al. demonstrated that the EBL-based approach allows fabricating large arrays of nanostructures with high reproducibility,^13^ their approach also comes with some limitations. Most evidently, the method does not allow the fabrication of hybrid material systems. Furthermore, the EBL process is based on the negative-tone resist hydrogen-silsesquioxane (HSQ) that provides very high resolution below 10 nm but requires process chemicals like tetramethylammonium hydroxide (TMAH) and hydrofluoric acid (HF) that are corrosive for many metals. Since this limits the flexibility of the fabrication process and consequently the broad and optimal applicability of AiB platforms, we developed a high-resolution two-step overlay EBL process (see Figure 3) that enables the use of various metals and hybrid systems.

For the experimental validation of the computational findings, we fabricated 10 × 10 arrays of isolated Au-BNAs, Au-Au-AiBs, and Au-Al-AiBs with an edge-to-edge period of 3 μm. In all three cases, the Au-BNA had a nominal length of *l* = 55 nm and a gap size of *g* = 20 nm. The real BNA length was found to be around *l* = 65–70 nm due to the proximity effect during the EBL process, thus matching the geometrical parameters of the simulations. For the AiB platforms, nominal aperture diameters of *d* = 400, 450, ..., 800 nm were used. As shown in Figure S4 and associated text of the Supporting Information, the measured diameter was within Δ*d* = ±10 nm of the nominal diameter and the overlay accuracy of around Δ*r* = 35 nm barely affected the computed excitation intensity enhancement.

**Figure 4:**
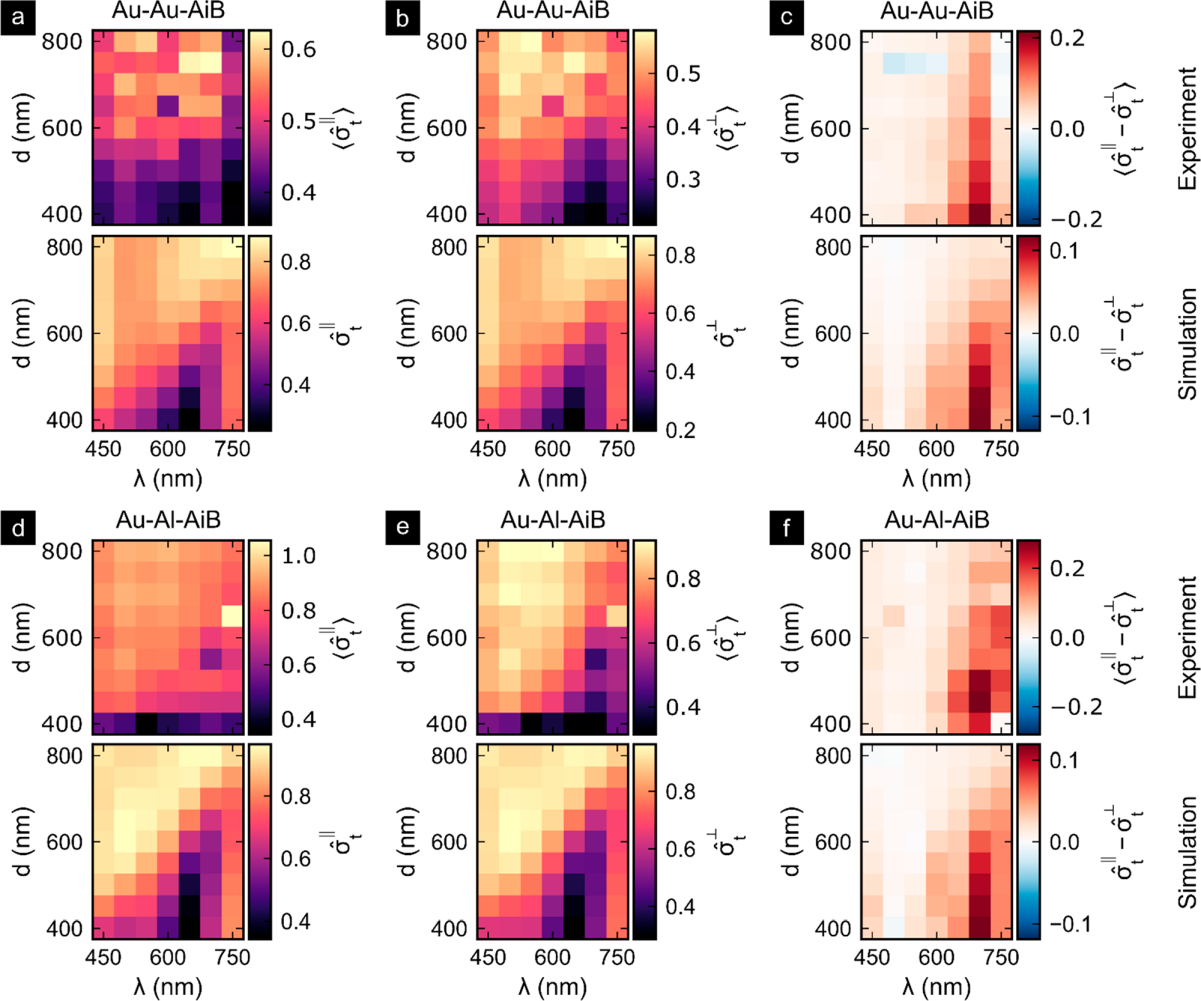
Experimental and simulated spectral transmission cross sections of the Au-Au-AiBs (a–c) and Au-Al-AiBs (d–f). The spectral transmission cross sections σ̂_*t*_ are normalized to the aperture area and measured for the incoming light being polarized parallel (a, d) and perpendicular (b, e) to the BNA axis. In (c, f) the differences of the transmission cross sections for parallel and perpendicular polarization are shown. The experimental data show the mean transmission cross sections over 40–64 nominally equivalent nanostructures in the arrays. The simulations are using illumination by a plane wave at normal incidence, whereas a NA = 1.34 illumination was used for the experimental data.

To experimentally validate the accuracy of the computational model and thus the diameter dependence of the computed excitation, emission, and SBR enhancement factors, we performed spectrally and polarization-resolved transmission measurements with the Au-Au-AiB and Au-Al-AiB arrays based on an approach developed by Payne et al.^38,39^Figure 4 shows the transmission cross sections for the incoming light being polarized parallel σ̂_t_^∥^ or perpendicular σ̂_t_^⊥^ to the BNA axis normalized to the respective nanoaperture area. The difference σ̂_t_^∥^ – σ̂_t_^⊥^ is also given to isolate the longitudinal Au-BNA resonance position. The experimental data are the average values ⟨...⟩ over the individual nominally identical nanostructures in the array, to reduce the influence of geometrical fluctuations. The employed FDTD model is similar to the model used in Figure 2 but was adapted to the changed experimental conditions. More details about the model and the calculation of the normalized transmission cross sections can be found in Figure S5 and associated text of the Supporting Information. For Au-Au-AiB and Au-Al-AiBs we found good agreement between experiment and simulation of the perpendicular transmission cross sections Figure 4b,e and the cross-section differences Figure 4c,f. In particular, the perpendicular component of Au-Al-AiB shows good agreement between the measured and simulated magnitudes. The magnitude of the experimental cross-section differences in Figure 4c,f are about twice the computed differences but agree well in the spectral peak position. The former explains the deviations observed in the parallel components in Figure 4a,d and could originate from the plane wave normal incidence illumination employed in the FDTD model in contrast to the high NA = 1.34 illumination geometry of the experiment. Effects of similar magnitude due to differences in the illumination geometry have been reported previously.^40^ Overall, there is good agreement in magnitude and spectral position of the transmission cross sections. These results support the assumption that the calculated excitation intensity enhancement factors are describing the nanostructures well.

We further performed time-correlated single-photon counting (TCSPC) measurements to assess the fluorescence enhancement factors provided by the different nanostructure platforms. For each of these measurements, Alexa Fluor 647 was embedded at different concentrations in an approximately 70 nm thick PMMA layer on top of glass coverslip substrates and excited with a λ_exc_ = 640 nm laser with a repetition rate of *f*_rep_ = 20 MHz. A first set of measurements was carried out on an unpatterned coverslip to determine the reference single-molecule fluorescence strength. The second set consisted of measuring the fluorescence signal as a function of time on different Au-BNAs, and the third and fourth sets of measurements recorded the fluorescence emission as a function of time on the Au-Au- and hybrid Au-Al-AiBs. For each spot measurement, a 60 s time trace was recorded. For this, the fluorescence emission was filtered with 37 nm wide band-pass filters centered around 676 nm in front of two single-photon avalanche diodes (SPADs) after passing a 50/50 beamsplitter. This configuration enabled recording not only the fluorescence time traces but also simultaneously the fluorescence decay curves and allowed determining the signal originating from a single molecule through blinking and bleaching events.

**Figure 5:**
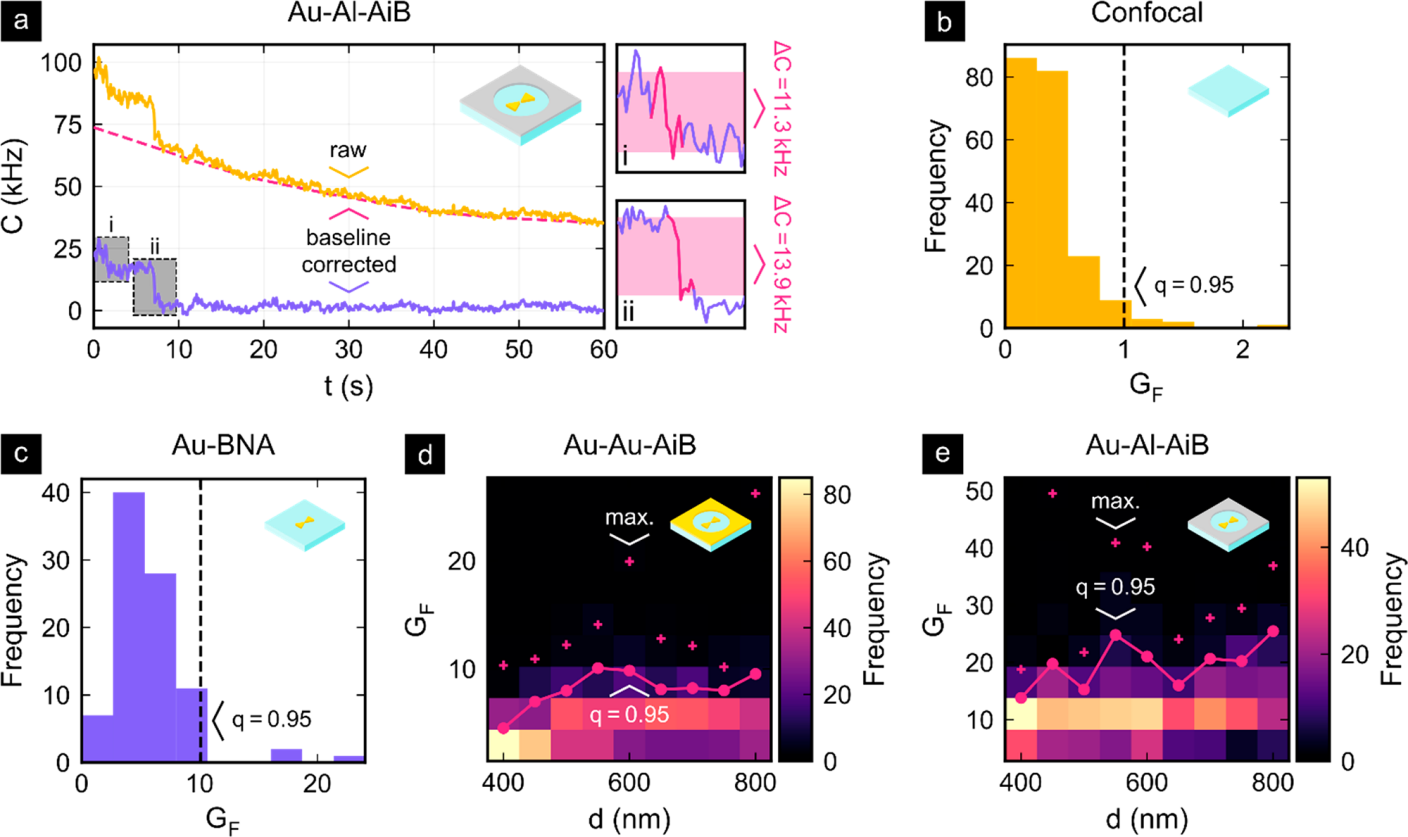
Fluorescence enhancement analysis of Alexa Fluor 647 embedded in PMMA for the confocal reference and the different nanostructure types. (a) Exemplary time trace *C*(*t*) from a 60 s spot measurement on Au-Al-AiB deduced from TCSPC data with a 100 ms binning time. The raw time trace was baseline corrected before detecting the blinking or bleaching step height Δ*C* of a single molecule through an automated algorithm. The fluorescence enhancement *G*_F_ is computed through the normalization of Δ*C* as described in the main text. (b) Histogram of the fluorescence enhancement for the confocal reference measurements. *G*_F_ = 1 was chosen to be at the *q* = 0.95 quantile of the confocal distribution. (c) Fluorescence enhancement histogram for the BNAs showing 10–22 × fluorescence enhancement (*q* = 0.95–1). (d) Diameter-dependent histogram of *G*_F_ for Au-Au-AiBs with the maximum enhancement (red crosses) and 0.95 quantile (solid line) overlaid. (e) Same as (d) but for the hybrid Au-Al-AiBs. While the Au-Al-AiBs provide a fluorescence enhancement of 26–50×, the Au–Au-AiBs provide a fluorescence enhancement more similar to that of Au-BNAs of 10–26×.

To determine the fluorescence enhancement, we applied a custom algorithm that detects count rate changes Δ*C* originating from fluorescence bleaching or blinking events in the baseline-corrected^41^ fluorescence time traces that have a 100 ms time binning. An exemplary time trace with the detected bleaching events is depicted in Figure 5a. Additional time traces and a detailed explanation of the algorithm can be found in Figures S6 and S7 and associated text of the Supporting Information. Having determined Δ*C*, the single-molecule fluorescence signal was calculated with the excitation power *p*_exc_ as
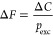
7for the confocal reference measurements and the different nanostructure types. The fluorescence enhancement
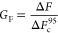
8was finally obtained by normalizing the single-molecule fluorescence signals Δ*F* by the *q* = 0.95 quantile of the confocal single-molecule fluorescence signals Δ*F*_C_^95^. The experiments were carried out with dye concentrations of 10 nM for the confocal measurements, 500 nM for the BNAs, and 2000 nM for both AiB types. These concentrations provided a good trade-off between increasing the signal-to-background ratio and the probability of finding an Alexa Fluor 647 molecule in the BNA gap region for each setting. As depicted in Figure 5, the fluorescence enhancement is between (c) 10 and 22× for the Au-BNAs, (d) 10 and 26× for the Au-Au-AiBs, and (e) 26 and 50× for the hybrid Au-Al-AiBs (*q* = 0.95–1). The ratios of the maximum experimental fluorescence enhancements are thus in good agreement with the simulated ratios. Indeed, the Au-Au-AiBs provide a slightly higher maximum fluorescence enhancement than the Au-BNAs whereas the hybrid Au-Al-AiBs provide about a 1.9× higher maximum fluorescence enhancement than the gold counterpart. We attribute the lower fluorescence enhancement factors and the absence of a clear nanoaperture diameter dependence in the experimental data as compared to the simulations to several reasons. Most importantly, the location and alignment of the static dyes in the BNA gap region induce a great spread in the observed fluorescence enhancement, concealing the rather subtle influence of the nanoaperture diameter and complicating the observation of the highest possible fluorescence enhancement factors. In addition, the accumulated effect of material defects, overlay offsets, or imperfect nanostructure geometries is expected to reduce the experimentally achievable fluorescence enhancement factors.

**Figure 6:**
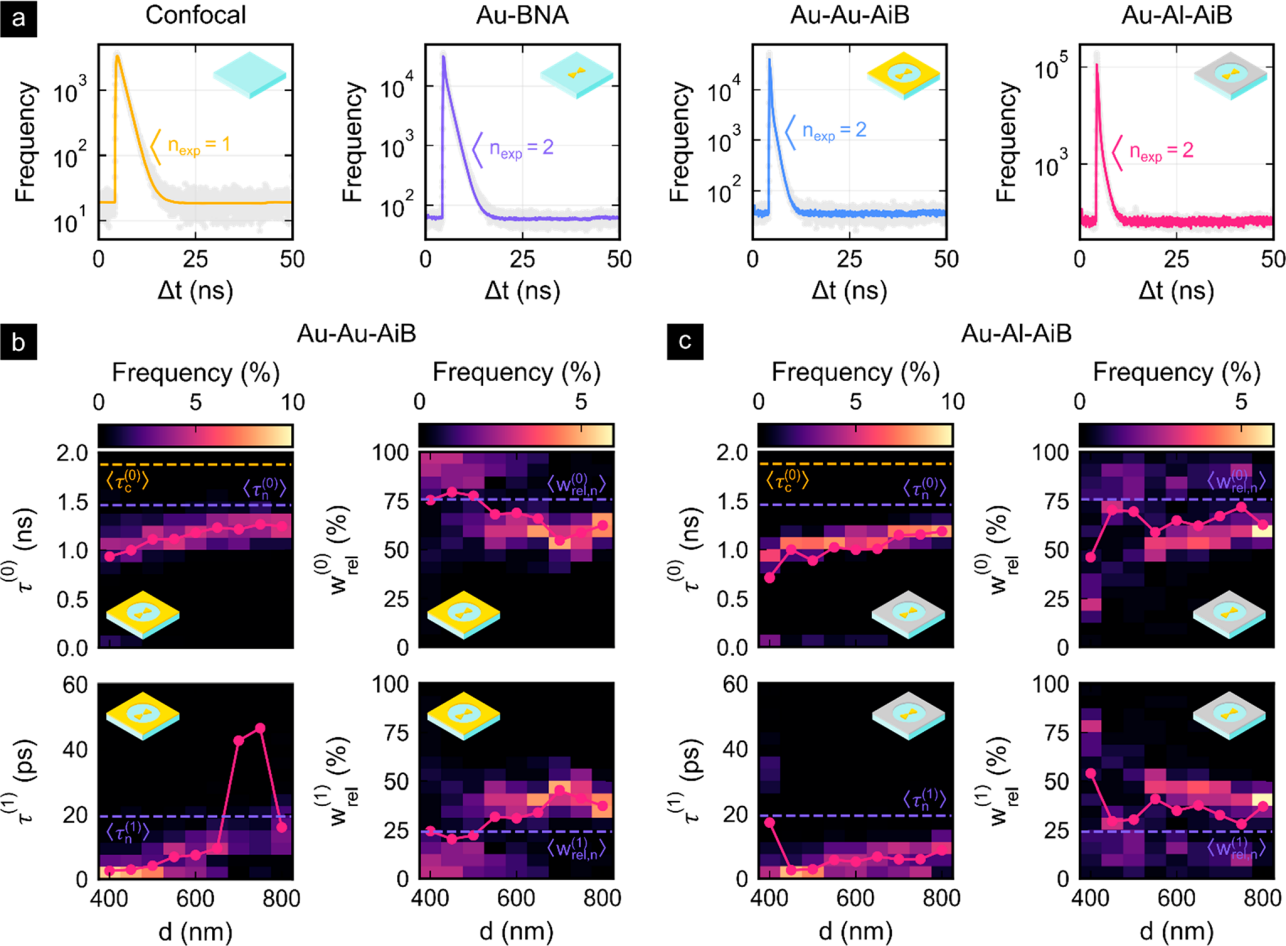
Fluorescence decay analysis of Alexa Fluor 647 embedded in PMMA for the confocal reference and the different nanostructure types. (a) Exemplary measured decay curves (light gray) with the corresponding reconvolution fits (colored) and number of exponentials *n*_exp_. (b) Decay times τ^(*i*)^ and relative weights *w*_rel_^(*i*)^ of the *i*th component for different aperture diameters of the Au-Au-AiB platform. The solid red lines indicate the averages for the Au-Au-AiBs, while ⟨τ^(*i*)^⟩ and ⟨*w*_rel_^(*i*)^⟩ indicate the average decay time and relative weights of the confocal reference (subscript c) and Au-BNA (subscript n). (c) shows the same as (b) but for the hybrid Au-Al-AiB platforms.

The decay curves shown in Figure 6 were acquired simultaneously with the fluorescence time traces and provide additional information about the decay channels. To extract the decay times τ^(*i*)^ and amplitudes *a*^(*i*)^ of the *i*th component the multiexponential temporal reconvolution fit
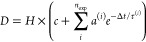
9was applied to each spot measurement. Here, *H* is the instrument response function (IRF, fwhm = 33 ps), *c* the *y*-offset, *n*_exp_ the number of exponential decays, and Δ*t* the time difference between the excitation pulse and emitted photon. The weights *w*^(*i*)^ = *a*^(*i*)^τ^(*i*)^ and relative weights *w*_rel_^(*i*)^ = *w*^(*i*)^/∑_*i*_^*n*_exp_^*w*^(*i*)^ are proportional to the number of photons in each fluorescent component. While a single-exponential decay was found to describe well the decay in the confocal reference measurements, a second faster component was required for the nanostructured platforms. As expected, the Purcell effect affects the lifetime of the fluorophores, especially reducing the lifetime in the BNA gap region of high LDOS. With increasing diameter, the decay times of both AiB types approach the average value of the BNA ⟨τ_n_^(0)^⟩ = 1.46 ns. This is expected, as the influence of the nanoaperture vanishes as the diameter approaches infinity. Furthermore, for almost all AiB diameters the weight of the short-lived component is above that of the BNAs. Only the weights at *d* = 450 nm and *d* = 500 nm of the Au-Au-AiB slightly deviate from this observation. This suggests that the aperture indeed ensures that more photons from dyes in the gap region are detected relative to the number of photons from the background. That the shorter lifetimes of both AiB types are mostly well below the average BNA lifetime ⟨τ_n_^(1)^⟩ = 19 ps additionally suggests an increased fluorescence emission of dyes in the gap region of AiBs. It should be noted that the short lifetimes are much below the fwhm of the IRF, inducing more significant inaccuracies in the fitting procedure. Comparing the Au-Au-AiB and Au-Al-AiB platforms, we found that most of the fast and slow lifetimes of the hybrid system are slightly shorter. Moreover, the weights of the fast component are higher for smaller diameters of the nanoaperture. In view of the higher fluorescence enhancement factors observed in Figure 5e, this supports the picture of an enhanced fluorescence emission and increased background screening provided by the hybrid Au-Al-AiB platforms.

## Conclusions

In this work, we demonstrated that hybrid Au-Al-AiB platforms can provide higher excitation intensity enhancement, fluorescence enhancement, and signal-to-background ratios as compared to isolated Au-BNAs and Au-Au-AiBs. We investigated computationally how AiB platforms can be optimized for a target wavelength range and demonstrated that controlling the nanoaperture diameter of AiBs allows finding a trade-off between maximum fluorescence enhancement and background reduction. For hybrid Au-Al-AiBs optimized for high signal enhancement, we computationally found that the excitation intensity enhancement can be 3.4× higher than for isolated Au-BNAs (Δ*G*_I_ = 2.4) and 1.3× higher than for Au-Au-AiBs (Δ*G*_I_ = 1.6). For hybrid Au-Al-AiBs optimized toward high SBR, we found an up to 13× higher SBR than for Au-BNAs and an 1.3× higher SBR than for Au-Au-AiBs. We expect that significantly higher SBR gains are possible for samples with large *z*-extent, as the simulations were carried out for background emitters in a 70 nm thin PMMA layer. To experimentally validate the superior performance of hybrid Au-Al-AiB platforms, we introduced a two-step EBL overlay process that provides more flexibility in terms of material selection and combinations without sacrificing the large-area capabilities. We showed that this approach allows fabricating Au-Au-AiBs and hybrid Au-Al-AiBs reproducibly and foresee a much wider range of possible material combinations paving the way toward a variety of hybrid plasmonic systems. Through spectrally resolved transmission cross-section measurements we demonstrated experimentally the diameter dependence of the resonance position and strength of the AiBs and validated that the employed computational model is accurate, suggesting that the predicted excitation enhancement factors are valid. Finally, we investigated the fluorescence enhancement factors provided by the different nanostructures through TCSPC spot measurements of Alexa Fluor 647 embedded in a 70 nm PMMA layer and found that the fluorescence enhancement of hybrid Au-Al-AiBs is about 2.3× higher than that of Au-BNAs and 1.9× higher than that of Au-Au-AiBs, being in good agreement with the simulations. The enhanced fluorescence emission and reduced background are supported by a fluorescence decay analysis exhibiting strongly decreased fast lifetime components for both AiB platforms and reduced strength of slower decay components attributed to the fluorescence background. The hybrid Au-Al-AiBs predominantly show slightly decreased lifetimes and amplitudes of the long-lived component that are attributed to the fluorescence background. Furthermore, the decay analysis demonstrated a vanishing influence of the nanoaperture for increasing diameters.

Having demonstrated the benefits of the hybrid Au-Al-AiB platform, it now awaits application to biologically relevant systems such as cell secretion^42^ or cell membrane^15^ studies. The presented fabrication process enables using hybrid AiBs as nanophotonic biosensor platforms for a wide range of biological interactions, as it allows accessing a large geometrical parameter space and employing a wider range of plasmonic materials, providing a broad tunability of the resonance position. Composite plasmonic nanostructures made of silver and aluminum could be deployed for multicolor fluorescence detection.^43−46^ In this regard, the use of ultrathin passivation layers will gain importance to stabilize the plasmonic materials and protect the cells from cytotoxic interactions.^47,48^ Especially, large hybrid AiB arrays will be crucial for biosensing applications involving the study of multiple species via multicolor labeling. Here, the AiB arrays will enable rapid acquisition of statistically relevant data through camera-based multiplexed readout following an approach similar to that recently shown for diffusion studies of single-color labeled molecules in living cells using traditional Au-Au-AiBs.^49^ Moreover, applying planarization procedures such as template stripping to the hybrid AiB platforms could extend their applicability to biological samples that require flat substrates.^13^ The platform also holds great potential for label-free sensing applications such as SERS^31−33^ or SECARS^34^ that could not only benefit from reduced background signals but particularly from the strong enhancement factors due to their nonlinear nature and low scattering cross sections.^35^

## Methods

### Nanostructure Fabrication

Borosilicate-crown glass (BK7) coverslips (#1.5 or #2) were cleaned by 15 min sonication in acetone followed by rigorous rinsing with isopropanol and Milli-Q water. Subsequently, the coverslips were nitrogen blow-dried and put on a hot plate for 3 min at 155 °C. For the first EBL step, the positive-tone resist AR-P 6200.04 (Allresist) was spin-coated for 1 min at 4000 rpm and then baked on a hot plate for 2 min at 155 °C. The conductive polymer Espacer 300Z (Showa-Denko) was spin-coated for 1 min at 5000 rpm on top of the resist to prevent charging due to the insulating substrate. For the exposure, a 30 kV Raith Elphy Plus system was used with the smallest electron beam aperture (30 μm, spot size 1) and a step size of 5 nm. The BNA patterns were exposed at a dose of 422.5 μC/cm^2^ and larger features such as alignment markers and labels at a lower dose of 130 μC/cm^2^. After the exposure, the conductive polymer was removed by a 15 s bath in Milli-Q water and nitrogen blow-drying. The resist was then developed for 2 min in AR 600-546 (Allresist) and rigorously rinsed with isopropanol and Milli-Q water before nitrogen blow-drying. A 1 nm chromium adhesion layer followed by a 50 nm gold layer were deposited using a Leybold Univex 350 evaporator. The lift-off was done with an approximately 2 h bath in AR 600-71 (Allresist) followed by 15 min sonication. Afterward, the sample was immediately rinsed with isopropanol and Milli-Q water and nitrogen blow-dried, concluding the BNA fabrication. Before the second EBL step for the AiB fabrication, the coverslips were baked on a hot plate for about 3 min at 155 °C for better resist adhesion. The negative-tone resist AR-N 7520.073 (Allresist) was then spin-coated for 1 min at 2000 rpm and baked on a hot plate for 2 min at 155 °C. The conductive polymer was spin-coated as detailed above. The nanoaperture pattern was exposed with the same EBL system and exposure parameters of the first step but with a spot size of 2 and a dose of 300 μC/cm^2^. An automatic alignment procedure that detects the center coordinate of eight markers (four to detect the *x* and *y* positions, respectively) was used to fully automate the exposure and overlay alignment process. After the exposure, the conductive polymer was removed as detailed before. The resist was then developed for 2 min in AR 300-26 (Allresist) with a 1:2 dilution in Milli-Q water. The development was stopped by rinsing with Milli-Q water before nitrogen blow-drying. A 1 nm chromium adhesion layer followed by a 50 nm gold layer or a 50 nm aluminum layer was deposited for the Au-Au-AiB or Au-Al-AiB, respectively, using the evaporator. The lift-off was done with an immediate 30 min sonication in AR 600-71 (Allresist). Afterward, the sample was rinsed with isopropanol and Milli-Q water and nitrogen blow-dried.

### Transmission Measurements

To prepare the samples for the transmission measurements, the nanostructured sides of the coverslips were covered with 30 μL of immersion oil (*n* = 1.518) and attached to a microscope slide. The coverslip and microscope slide were compressed to achieve an immersion oil thickness of only a few micrometers, and the edges were sealed with nail varnish before being mounted in the microscope. The transmission measurements were carried out with a Nikon Ti-U inverted wide-field microscope. A 100 W halogen lamp with 40 nm band-pass filters centered at 450, 500, ..., 750 nm was used as the illumination source. The light passed through a NA = 1.34 oil-immersion condenser (Nikon MEL41410) and was collected with a 100×, NA = 1.45 oil-immersion objective (Nikon MRD00405) with a 1× tube lens. A rotatable linear polarizer was inserted in front of the condenser to control the polarization of the excitation light. The transmission images were acquired with a a scientific CMOS (sCMOS) camera (PCO Edge 5.5) on top of a 10 × 10 array of nominally equal nanostructures with a resolution of 2560 × 2160 pixels. The transmission cross sections were calculated from an average of 256 individual acquisitions per array and reference images taken with empty coverslips.

### Fluorescence Measurements

The fluorescence time traces and decay curves were computed from the same TCSPC data that were recorded with a commercial MicroTime 200 (PicoQuant) setup attached to an Olympus IX71 microscope body. For the excitation, a linearly polarized laser diode (LDH D-C-640) was operated with a repetition rate of 20 MHz at λ_exc_ = 640 nm and focused and collected with an Olympus UPlanSApo 60×, NA = 1.2 water-immersion objective. The collected light was detected with two SPADs (PDM Series, PicoQuant) after passing through a dichroic mirror, a 75 μm confocal pinhole, a 50/50 beamsplitter, and two emission band-pass filters (FF01-676/37-25, Semrock) in front of each of the SPADs. A three-axis piezoelectric stage (P-733.2, Physik Instrumente) allowed the precise localization of the nanostructures. The commercial software SymPhoTime 64 (PicoQuant) was used to control the experiment and manage the data acquisition and storage. The analysis and visualization of the binary time-tagged time-resolved data were carried out using custom-made Python scripts. For the preparation of the samples, Alexa Fluor 647 Carboxylic Acid (Molecular Probes) was diluted in PMMA (AR-P 639.04, Allresist) to the desired concentration and spin-coated onto the sample for 1 min at 8000 rpm. Afterward, the sample was baked on a hot plate for 3 min at 155 °C. The IRF was determined by detecting the excitation light backscattered from an empty coverslip during a 60 s TCSPC measurement with removed emission filters and at the lowest laser power to not overexpose the SPADs.

## Supporting Information Available

FDTD simulations of the optimal Au-BNA length for Au-Au- and Au-Al-AiBs, FDTD simulations of the cavity modes in Al and Au apertures, explanation of the FDTD simulation setups, SEM images of Au-Au- and Au-Al-AiBs with offset and diameter studies, explanation of the experimental and computational transmission cross-section determination, detailed discussion of the blinking and bleaching detection algorithm, exemplary time traces for all nanostructure types and the confocal reference, exemplary decay curves for all nanostructure types and the confocal reference, and power dependence of the fluorescence signal (PDF)
