# Supplementary material for: Hybrid Plasmonic Nanostructures for Enhanced Single-Molecule Detection Sensitivity
Source: ACS Nano. 2023 Apr 3;17(9):8453–64. doi: 10.1021/acsnano.3c00576 (PMC10173688; doi:10.1021/acsnano.3c00576)
Supplement: Supplementary file 1 — nn3c00576_si_001.pdf [file nn3c00576_si_001.pdf]

Supporting Information for

Hybrid plasmonic nanostructures for enhanced single molecule detection sensitivity

Ediz Kaan Herkert[†*], Domenica Romina Bermeo Alvaro[†], Martina Recchia[‡], Wolfgang W. Langbein[‖], Paola Borri[‡], Maria F. Garcia-Parajo[†§]

† ICFO-Institut de Ciencies Fotoniques, The Barcelona Institute of Science and Technology, 08860 Castelldefels (Barcelona), Spain

‡ School of Biosciences, Cardiff University, Museum Avenue, CF10 3AX Cardiff, United Kingdom

‖ School of Physics and Astronomy, Cardiff University, The Parade, Cardiff CF24 3AA, United Kingdom

§ ICREA, Pg. Lluís Companys 23, 08010 Barcelona, Spain

***Corresponding Author:** ediz.herkert@icfo.eu

Keywords: optical nanoantennas, plasmonic biosensing, electron beam lithography, hybrid materials, plasmonics

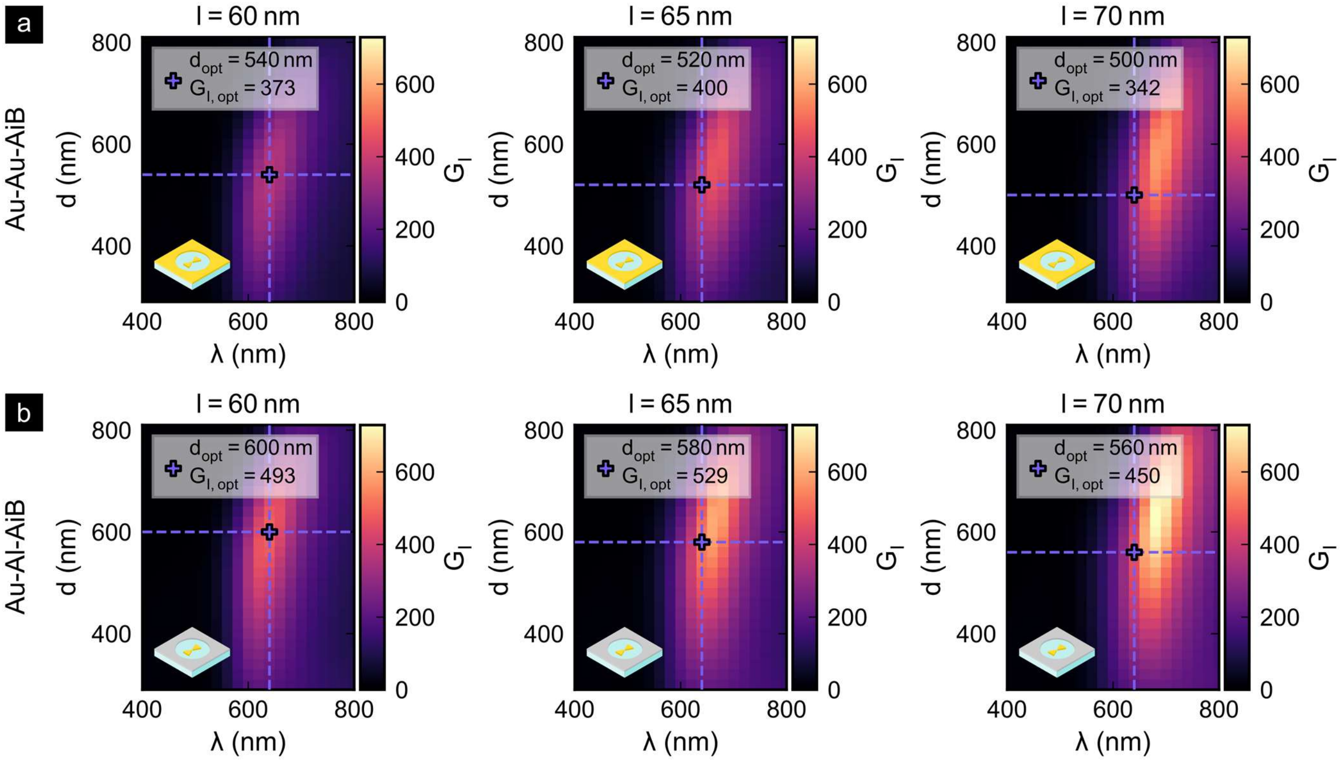

Figure S1. FDTD simulations of the antenna-in-box (AiB) excitation intensity enhancement G_I in dependence on the aperture diameter for different gold bowtie nanoantenna (Au-BNA) lengths l of Au-Au-AiBs (a) and Au-Al-AiBs (b). The dashed lines indicate the optimal diameter d_{opt} maximizing the excitation intensity enhancement at $\lambda_{\mathrm{exc}} = 640\ \mathrm{nm}$. All simulations are performed with a y-polarized total-field scattered-field (TFSF) plane wave source and perfectly matched layer (PML) boundaries with the intensities being computed in the hotspot center at $(0, 0, 26)\ \mathrm{nm}$.

Section 1: Simulation of the optimal Au-BNA length of AiBs

Two sets of simulations were carried out to determine the gold bowtie nanoantenna (Au-BNA) length l and aperture diameter d that maximize the excitation intensity enhancement G_I of the gold/gold antenna-in-box (Au-Au-AiB) and gold/aluminum antenna-in-box (Au-Al-AiB) designs. The first set of simulations was carried out for the Au-Au-AiB optimization shown in Figure S1 (a) and yielded maximal excitation intensity enhancement at $\lambda_{\mathrm{exc}} = 640\ \mathrm{nm}$ for $d = 520\ \mathrm{nm}$ and $l = 65\ \mathrm{nm}$. Figure S1 (b) shows that Au-Al-AiBs provide maximal excitation intensity enhancement at the same Au-BNA length of $l = 65\ \mathrm{nm}$ but a slightly larger aperture diameter of $d = 580\ \mathrm{nm}$. Based on these results, $l = 65\ \mathrm{nm}$ was chosen for our study as Au-BNA length for the isolated Au-BNA, the Au-Au-AiB, and the Au-Al-AiB.

Remarkably, Figure S1 also illustrates that maximal excitation intensity enhancement is not achieved when the excitation wavelength λ_{exc} and the localized surface plasmon resonance (LSPR) wavelength λ_{res} coincide ($\lambda_{\mathrm{exc}} = \lambda_{\mathrm{res}}$ at about $l = 60\ \mathrm{nm}$) but for Au-BNA lengths that support a resonance wavelength slightly red-shifted to the excitation wavelength. We attribute this to mainly two reasons. Firstly, longer Au-BNA lengths induce a red-shift of the

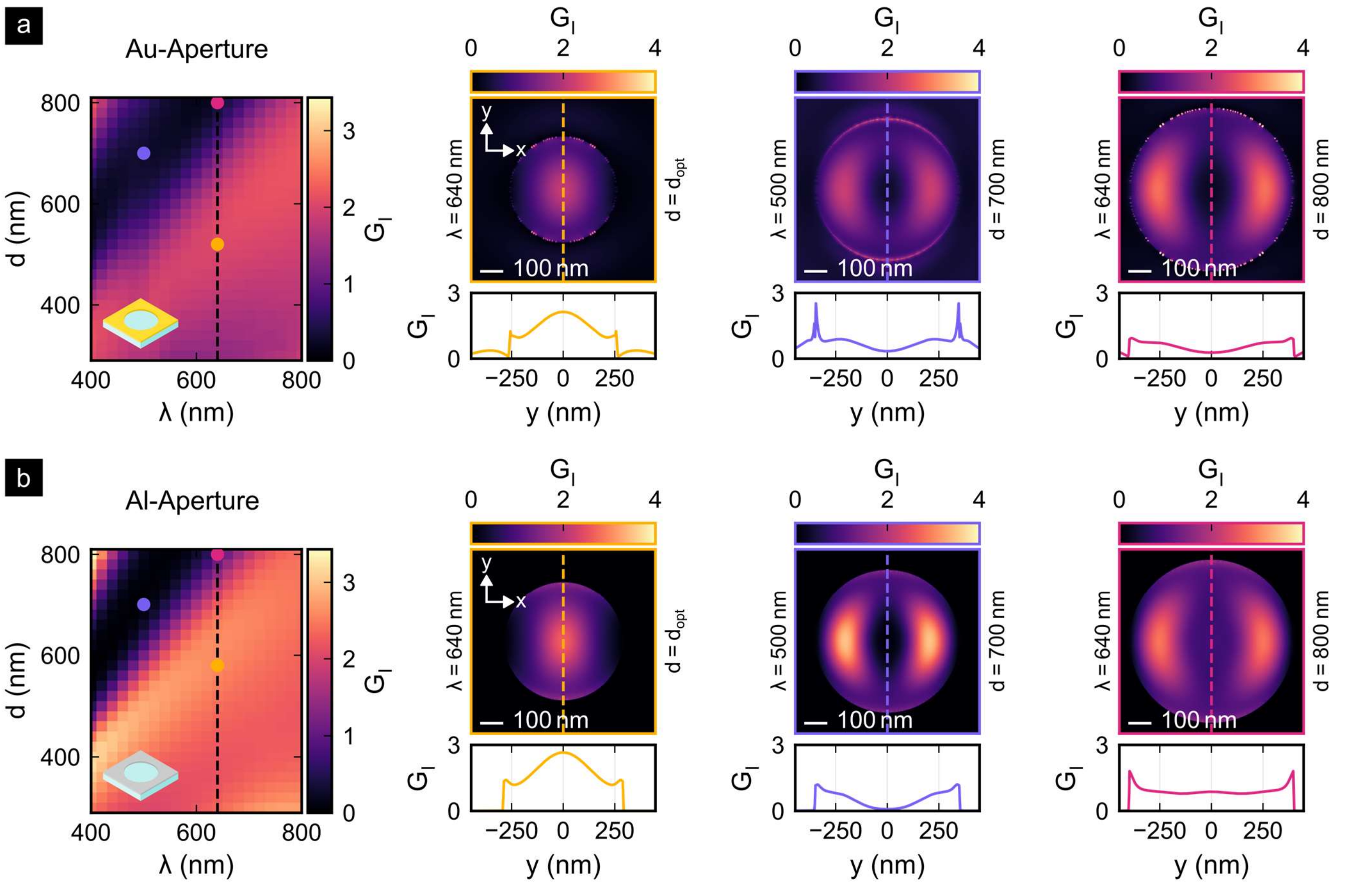

Figure S2. FDTD simulations of the cavity modes in Au- and Al-apertures. The diameter dependent excitation intensity enhancement $G_I(\lambda, d)$ is computed in the aperture center $(x, y, z) = (0, 0, 26a)$ nm and illustrates the presence of cavity modes in the (a) Au- and (b) Al-apertures. The field distributions in the xy-plane and along the y-axis show the formation of the fundamental mode at $(\lambda, d) = (640\text{ nm},\ d_{\text{opt}})$ (yellow) and the first harmonic at $(\lambda, d) = (640\text{ nm},\ 800\text{ nm})$ (red) and $(\lambda, d) = (500\text{ nm},\ 700\text{ nm})$ (purple) changing the field overlap with the BNA in the case of AiBs. The dashed lines in the xy-planes indicate the intersecting lines corresponding to the plots below. The xy-plane plots are clipped to $G_I = 4$ to improve the visibility of relevant areas despite the high intensities at the aperture rim introduced by the finite mesh size. The optimal diameter d_{opt} is (a) 520 nm or (b) 580 nm, respectively, according to the data presented in the main text. All simulations are performed with a y-polarized TFSF plane wave source and PML boundaries.

resonance but also increase the dipole moment of the resonator and thus the overall resonance amplitude. Secondly, the increased size of the Au-BNA provides a better spatial overlap with the fundamental cavity mode of the aperture shown in Figure S2 enabling a more efficient excitation of the Au-BNA LSPR. The finite-difference time-domain (FDTD) simulation layouts used for the optimization shown in Figure S1 are the same as for Figure 2 (a, b) of the main text and are detailed in Section 3: of the supporting information.

Section 2: Simulation of the cavity modes

As mentioned in the main text, the increased excitation intensity of the AiBs is due to the coupling of the aperture cavity modes with the Au-BNA resonance. That this coupling of the

cavity to the nanoantenna is indeed the driving factor for the higher excitation intensity of AiBs becomes clear when considering the intensity profiles in the xy-plane shown in Figure S2 for an Au- (a) and an Al-aperture (b). In both cases, the fundamental cavity mode occurs when the aperture diameter corresponds to the optimal AiB diameter d_{opt} maximizing the AiB excitation intensity enhancement. This is because the fundamental mode provides good spatial overlap with the Au-BNA and thus efficient LSPR excitation. This is in stark contrast to the first harmonic, where the excitation intensity is close to zero at the center of the aperture, as seen in Figure S2 for $(\lambda, d) = (640\,\mathrm{nm},\ 800\,\mathrm{nm})$ and $(\lambda, d) = (500\,\mathrm{nm},\ 700\,\mathrm{nm})$.

The very similar dependence of the aperture excitation intensity enhancement $G_I(\lambda, d)$ shown in Figure S2 and the relative AiB excitation intensity enhancement $\Delta G_I(\lambda, d)$ shown in the central row of Figure 2 (a, b) in the main text further emphasizes that the coupling of cavity modes to the Au-BNA LSPR is the key factor for the increased excitation intensity enhancement.

Section 3: Simulation of the excitation intensity and fluorescence enhancement

Two types of FDTD simulation templates were used to compute the excitation intensity enhancement and the fluorescence enhancement together with the signal-to-background ratios. Figure S3 depicts the refractive index distributions in the xy- and yz-plane as pseudocolor plot. For all simulations a $70\,\mathrm{nm}$ PMMA layer was defined as superstrate. For the AiB platforms the thickness of the PMMA layer was either defined from the top surface of the BK7 layer (b, c) or from the top surface of the metal film (e, f). This was done to allow for better comparison amongst the results obtained in (a – c) and (d – e) as will be discussed below.

The gold bowtie nanoantenna (Au-BNA) consisted of $50\,\mathrm{nm}$ gold on top of $2\,\mathrm{nm}$ chromium with a length of $l = 65\,\mathrm{nm}$, a gap size of $g = 20\,\mathrm{nm}$, an apex angle of $\alpha = 90°$, and an edge and corner curvature radius of $r_{\mathrm{c}} = 20\,\mathrm{nm}$. For the Au-Au-AiB, the aperture consisted of a $50\,\mathrm{nm}$ gold layer on top of a $2\,\mathrm{nm}$ chromium adhesion layer, whereas for the Au-Al-AiB the aperture consisted of a $50\,\mathrm{nm}$ aluminum (Al) layer. The excitation source was in all cases a y-polarized total-field scattered-field (TFSF) plane wave propagating in positive z-direction and placed $130\,\mathrm{nm}$ below the BK7/PMMA interface. Perfectly matched layers (PMLs) were used as simulation boundaries of the simulation volume of size $(1, 1, 0.35)\,\mu\mathrm{m}$ or

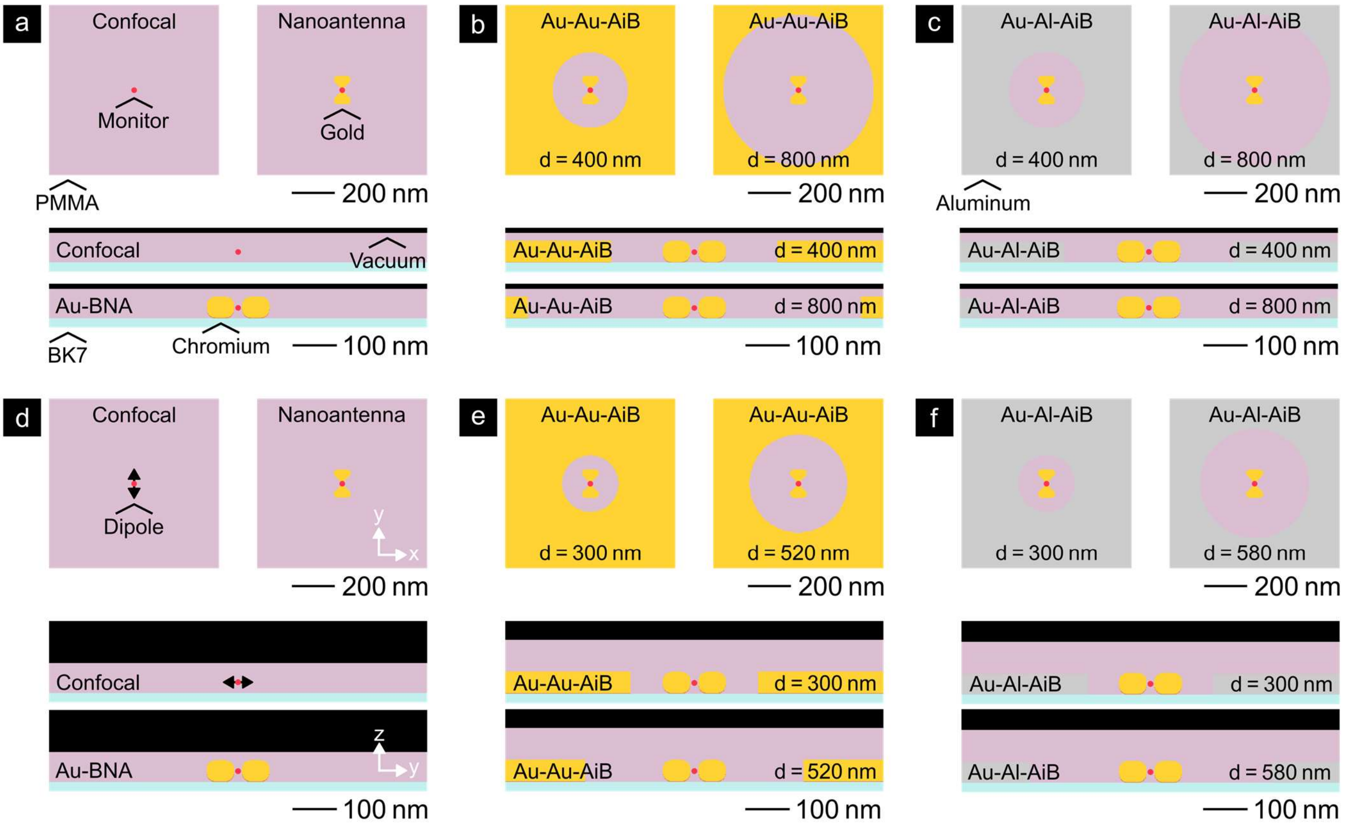

Figure S3. FDTD simulation setups reconstructed from the refractive index data of the simulation templates. (a – c) show the setups used to compute the spectral excitation intensity enhancement of the confocal reference, the Au-BNA, the Au-Au-AiB, and the hybrid Au-Al-AiB. The red dots indicate the location of the 0D monitor recording the electric fields. (d – f) show the setups used to compute the fluorescence enhancement and signal-to-background ratios. Here, the red dots indicate the location (d – f) and orientation (d) of the target dipole. The main difference between the setups in (a – c) and (d – f) is that for both AiBs the PMMA thickness of $70\ \mathrm{nm}$ is either measured from the upper BK7 surface (b, c) or from the upper metal film surface for better comparability of the fluorescence background (e, f). All simulations are performed with a y-polarized total-field scattered-field (TFSF) plane wave source and perfectly matched layer (PML) boundaries.

$(1, 1, 0.4)\ \mu\mathrm{m}$ for computing the excitation intensity or fluorescence enhancement, respectively.

The panels (a – c) show the templates used for the computation of the excitation intensity enhancement. The red dots in these plots indicate the location of the 0D monitor recording the spectrally resolved electric field vector $\boldsymbol{E}$ at the point $\boldsymbol{r} = (0, 0, 26)\ \mathrm{nm}$. This point was chosen as it corresponds to the geometrical center of the BNA gap. The excitation intensity enhancements provided by the BNA, the Au-Au-AiB, and the Au-Al-AiB were calculated by normalizing the absolute squared electric field vectors in the nanostructured setups by the absolute squared electric field vector in the confocal setup

$$G_I(\boldsymbol{r}) = \frac{|\boldsymbol{E}(\boldsymbol{r})|^2}{|\boldsymbol{E}_\mathrm{C}(\boldsymbol{r})|^2} \qquad (1)$$

For the computation of the fluorescence enhancement and signal-to-background ratio, the templates in panel (d – e) were employed. The fluorescence signal was obtained by placing a dipole aligned in y-direction in the gap center $\boldsymbol{r} = (0, 0, 26)\ \mathrm{nm}$. The fluorescence background was computed by incoherent addition of the fluorescence intensity radiated by $n = 40$ dipoles being randomly distributed and aligned within a rectangular $900\ \mathrm{nm} \times 900\ \mathrm{nm} \times 70\ \mathrm{nm}$ volume within the PMMA layer. However, placing the dipoles in a volume of $130\ \mathrm{nm} \times 150\ \mathrm{nm} \times 52\ \mathrm{nm}$ around the BNA was prohibited to avoid dipoles being placed within the BNA.

For better comparability of the background signals, the same dipole distribution and orientation was used for all simulation setups in (d – f) so that for the AiBs the $70\ \mathrm{nm}$ PMMA layer had to be shifted $52\ \mathrm{nm}$ (Au-Au-AiB) or $50\ \mathrm{nm}$ (Au-Al-AiB) in positive z-direction to be above the chromium/gold or aluminum layer, respectively. For all four setups (confocal, Au-BNA, Au-Au-AiB, Au-Al-AiB) two separate simulations were performed for each of the 41 dipoles to compute the excitation power at $\lambda_{\mathrm{exc}} = 640\ \mathrm{nm}$

$$p_{\mathrm{exc}}(\boldsymbol{r}, \lambda_{\mathrm{exc}}) = |\boldsymbol{n} \cdot \boldsymbol{E}(\boldsymbol{r}, \lambda_{\mathrm{exc}})|^2 \qquad (2)$$

at the site $\boldsymbol{r}$ of a dipole with orientation $\boldsymbol{n}$ and to determine the total power emitted by a dipole $p_{\mathrm{dip}}(\lambda_{\mathrm{emi}})$ together with the power radiated into the lower hemisphere $p_{\mathrm{rad}}^{-}(\lambda_{\mathrm{emi}})$ at $\lambda_{\mathrm{emi}} = 676\ \mathrm{nm}$. $p_{\mathrm{dip}}(\lambda_{\mathrm{emi}})$ was recorded through a $10\ \mathrm{nm} \times 10\ \mathrm{nm} \times 10\ \mathrm{nm}$ transmission box placed around each dipole measuring the net power flow out of that volume. A second $900\ \mathrm{nm} \times 900\ \mathrm{nm} \times 100\ \mathrm{nm}$ transmission box was placed just below the BK7/PMMA interface. The power flowing through the upper surface of this transmission box was neglected to obtain the net power flowing into the lower hemisphere $p_{\mathrm{rad}}^{-}(\lambda_{\mathrm{emi}})$. From these quantities the fluorescence power emitted into the lower hemisphere

$$p^{-} = p_{\mathrm{exc}}(\boldsymbol{r}, \lambda_{\mathrm{exc}}) \cdot \frac{p_{\mathrm{rad}}^{-}(\lambda_{\mathrm{emi}})}{p_{\mathrm{dip}}(\lambda_{\mathrm{emi}})} = p_{\mathrm{exc}}(\boldsymbol{r}, \lambda_{\mathrm{exc}}) \cdot \eta^{-} \qquad (3)$$

was computed for an intrinsic quantum efficiency of $\eta_0 = 1$ and with $p_{\mathrm{dip}}(\lambda_{\mathrm{emi}}) = p_{\mathrm{rad}}(\lambda_{\mathrm{emi}}) + p_{\mathrm{loss}}(\lambda_{\mathrm{emi}})$ being the sum of the total power radiated into the far-field $p_{\mathrm{rad}}(\lambda_{\mathrm{emi}})$ and the power dissipated through metal induced absorption losses $p_{\mathrm{loss}}(\lambda_{\mathrm{emi}})$. Here, η^{-} is the quantum efficiency for emission into the lower hemisphere. As for $\eta_0 = 1$ the fluorescence emission power enhancement and emission rate enhancement compared to a reference power and rate are related through [1]

$$\frac{p}{p_{\mathrm{ref}}} = \frac{\Gamma}{\Gamma_{\mathrm{ref}}} \qquad (4)$$

we computed the fluorescence enhancement of the target dye (subscript 0) relative to the confocal reference (subscript C) as

$$G_F = \frac{p_0^-}{p_{\mathrm{C},0}^-} \tag{5}$$

and the signal-to-background ratio for all $n_{\mathrm{tot}} = 40$ background dipoles as

$$\mathrm{SBR} = \frac{p_0^-}{\sum_{j=1}^{n_{\mathrm{tot}}} p_j^-} \tag{6}$$

For the different concentrations, the SBR is computed as the mean $\langle \mathrm{SBR} \rangle = \frac{1}{m}\sum_{i=1}^{m} \mathrm{SBR}_i$ of $m = n_{\mathrm{tot}}/n_{\mathrm{sub}}$ random subsets $i = 1, \ldots, m$ with $n_{\mathrm{sub}} = \{1, 2, 4, 10, 20, 40\}$ background dipoles. The dipole subsets are drawn such that at each concentration each of the 40 dipoles is present in exactly one subset. Defining x_{ij} as a boolean variable specifying whether a background dipole j is present in a subset i allows to show that the SBR enhancement

$$G_{\mathrm{SBR}} = \frac{\langle \mathrm{SBR} \rangle}{\langle \mathrm{SBR_C} \rangle} = \frac{p_0^-}{p_{\mathrm{C},0}^-} \cdot \frac{\frac{1}{m}\sum_{i=1}^{m}\sum_{j}^{n_{\mathrm{tot}}} x_{ij} \cdot p_{\mathrm{C},j}^-}{\frac{1}{m}\sum_{i=1}^{m}\sum_{j}^{n_{\mathrm{tot}}} x_{ij} \cdot p_j^-} = G_F \cdot \frac{\sum_{j=1}^{n_{\mathrm{tot}}} p_{\mathrm{C},j}^-}{\sum_{j=1}^{n_{\mathrm{tot}}} p_j^-} \tag{7}$$

is independent of the choice of subsets i and thus the given concentration.

Section 4: Overlay offset and diameter mismatch analysis

Based on the scanning electron microscope (SEM) images in Figure S4 (a, b) the real nanoaperture diameters d_r and the offsets Δr between the nanoaperture and BNA centers were measured. For this, circles were manually fitted to the circumference of the nanoaperture and rectangles to the bounding box of the BNA. The SEM scale bar was used to convert fitted sizes in pixels to nanometers. To compute the overlay offsets, the center-to-center distances between the fitted circles and rectangles were measured. This was done for each set of diameters containing the nine displayed nanostructures to compute the offset distribution Δr and the average difference between the real diameter d_r and the nominal diameter d_n that was specified in the layout file of the electron beam lithography (EBL) system.

We found an overlay accuracy of $30\ \mathrm{nm}$ for the Au-Au-AiB (c) and $36\ \mathrm{nm}$ for the Au-Al-AiB (d) with a precision of $10\ \mathrm{nm}$ for both. The real nanoaperture diameter was found to be within $\pm 10\ \mathrm{nm}$ of the nominal diameter. The panels (e, f) show FDTD simulations that were

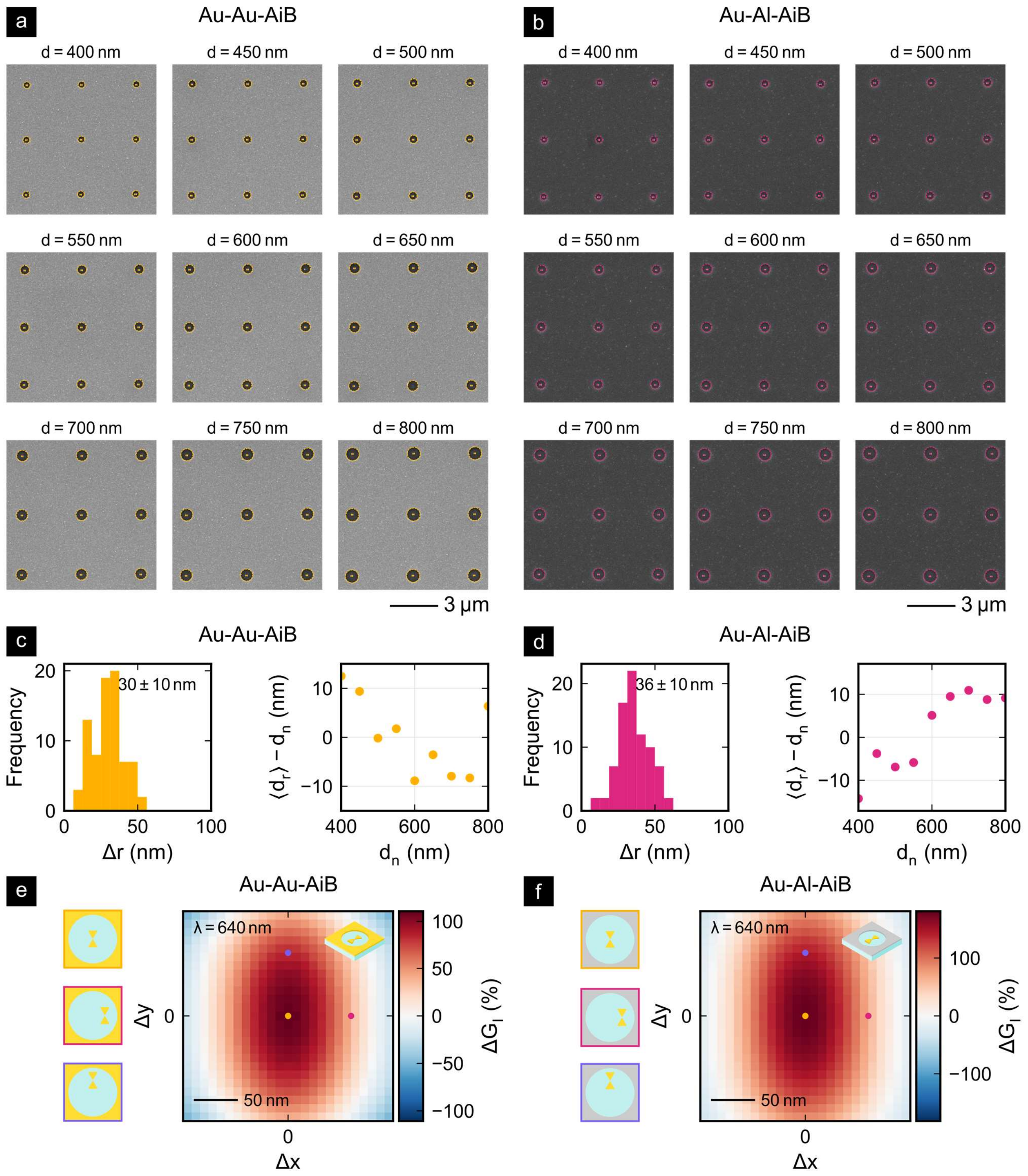

Figure S4. (a, b) show SEM images of the Au-Au- and Au-Al-AiBs for different nominal diameters with manually fitted circles and boxes overlaid that were used for the statistical analysis in (c, d). The distribution of the offsets Δr between the nanoantenna and nanoaperture centers and the mean deviations between real and nominal diameters d_r and d_n are shown in (c) for the Au-Au- and in (d) for the Au-Al-AiB platforms. The FDTD simulations of the relative excitation intensity enhancement ΔG_I for different center-to-center offsets $\boldsymbol{\Delta r} = (\Delta x,\ \Delta y)$ are shown in (e) for Au-Au- and in (f) for Au-Al-AiBs.

carried out to estimate the influence of the overlay offset $\boldsymbol{\Delta r} = (\Delta x, \Delta y)$ on the excitation intensity enhancement. Here, the diameters of the Au-Au-AiB and Au-Al-AiBs were $d_\mathrm{opt} = 520\ \mathrm{nm}$ and $d_\mathrm{opt} = 580\ \mathrm{nm}$, respectively, optimizing the excitation intensity. The relative

excitation intensity enhancement was defined similar to the main text as the relative difference between the excitation intensity enhancement of the AiB and the BNA

$$\Delta G_I = \frac{G_I^{\mathrm{AiB}} - G_I^{\mathrm{BNA}}}{G_I^{\mathrm{BNA}}} \tag{8}$$

The results show that the excitation intensity enhancement of the AiBs is above the one of BNAs for overlay offsets up to about $100\,\mathrm{nm}$. Furthermore, the excitation intensity enhancement was found to be more susceptible to offsets Δx perpendicular to the excitation polarization. The hybrid Au-Al-AiB was found to be slightly more robust against overlay offsets, which is related to the slightly larger nanoaperture diameter.

Section 5: Determination of the transmission cross-sections

Widefield transmission imaging was performed to experimentally determine the transmission cross-sections. The field of view (FOV) was chosen such that it fully contained a 10×10 array of AiBs of equal fabrication parameters. On each array, the average of 256 individual frames were taken for each excitation wavelength range of $40\ \mathrm{nm}$ width centered at $\lambda_{\mathrm{exc}} = 450, 500, \ldots, 750\ \mathrm{nm}$ and for two linear excitation polarization directions (along and across the BNA) at the back focal plane of the $\mathrm{NA} = 1.34$ condenser. The background corrected average image $I(x, y)$ was transformed to the transmission image

$$t(x, y) = \frac{I(x, y)}{\mathrm{I_{ref}}(x, y)} \tag{9}$$

with $\mathrm{I_{ref}}(x, y)$ being a reference image taken on an empty coverslip. For the analysis, two regions were defined. A foreground region

$$A_{\mathrm{fg}} = \pi \cdot r_{\mathrm{fg}}^2 = \pi \left(\frac{3 \cdot \lambda_{\mathrm{exc}}}{2 \cdot \mathrm{NA}} + r \right)^2 \tag{10}$$

for a disk with a radius r_{fg} centered at the peak of $I(x, y)$ indicating the position of the nanostructure. Here, $\mathrm{NA} = 1.45$ is the numerical aperture of the objective and r is the radius of the AiB nanoaperture. The disk is surrounded by the background region, given by a concentric ring of inner radius r_{fg}, outer radius $2r_{\mathrm{fg}}$, and area

$$A_{\mathrm{bg}} = 3\pi \cdot r_{\mathrm{fg}}^2. \tag{11}$$

The local background transmission

$$t_{\mathrm{bg}} = A_{\mathrm{bg}}^{-1} \int_{A_{\mathrm{bg}}} t(x, y)\, dA \tag{12}$$

was taken into account to determine the transmission cross-section given by

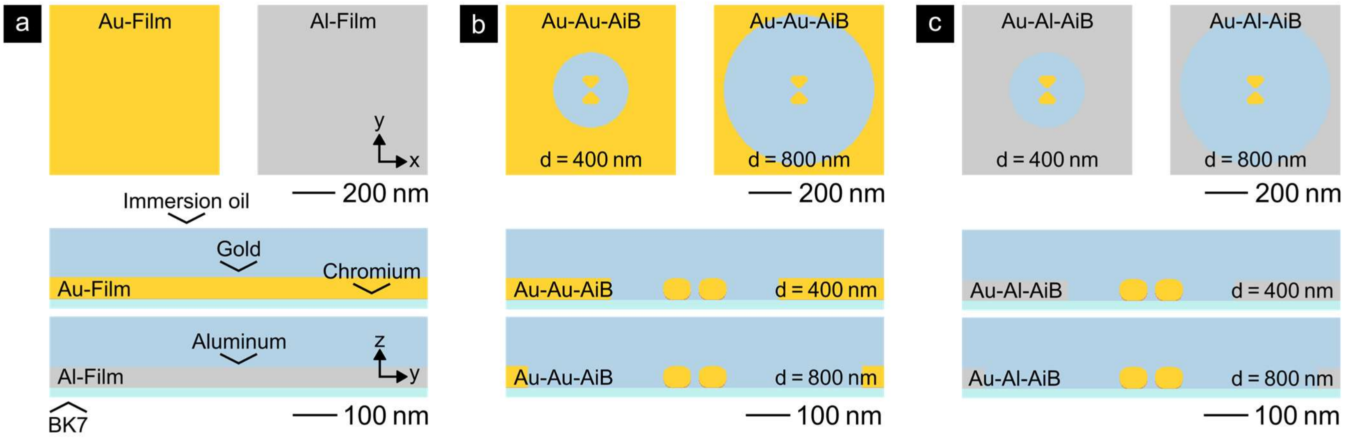

Figure S5. FDTD simulation setups reconstructed from the refractive index data of the simulation templates. (a – c) show the setups used to compute the transmission cross-section of the closed films, the Au-Au-AiB, and the hybrid Au-Al-AiB. All simulations are performed with a x- or y-polarized plane wave source and perfectly matched layer (PML) boundaries.

$$\sigma_{\mathrm{t}} = \int_{A_{\mathrm{fg}}} t(x,y) - t_{\mathrm{bg}}\, dA \tag{13}$$

which was then normalized by the area of the aperture, to yield the normalized transmission cross-section

$$\hat{\sigma}_{\mathrm{t}} = \frac{\sigma_{\mathrm{t}}}{\pi \cdot r^2} \tag{14}$$

for each excitation wavelength and polarization. This procedure [2] was implemented as ImageJ macro so that the transmission cross-sections of all nanostructures in the transmission image were returned individually allowing to compute the average normalized transmission cross-section for each nanoaperture diameter. Nanostructures that were too close to the image border to calculate the local background were dismissed.

A third set of FDTD simulations was set up to simulate the transmission cross-sections. The simulation setups were similar to the ones described in Section 3: but adapted to the experimental conditions of the transmission cross-section measurements. As depicted in Figure S5 (a – c), an immersion oil superstrate was used and additional simulations with closed aluminum and gold films were carried out. The simulation volume was chosen to be $(2, 2, 0.4)\ \mu\mathrm{m}$. Furthermore, the polarized plane wave was injected in negative z-direction from $130\ \mathrm{nm}$ above the BK7/metal interface and the transmitted power p_t measured with a $1500\ \mathrm{nm} \times 1500\ \mathrm{nm}$ monitor $100\ \mathrm{nm}$ below the BK7/metal interface. The uncorrected transmission cross-sections

$$\tilde{\sigma}_t = \frac{p_{\mathrm{t}}}{I_{\mathrm{exc}}} \tag{15}$$

were computed using the excitation intensity I_{exc} for the two closed films and AiBs. The corrected transmission cross-sections were then determined subtracting the finite transmission of the metal films, $\tilde{\sigma}_t^{\mathrm{F}}$, from the corresponding uncorrected transmission of the AiBs $\tilde{\sigma}_t^{\mathrm{AiB}}$, yielding

$$\sigma_t = \tilde{\sigma}_t^{\mathrm{AiB}} - \tilde{\sigma}_t^{\mathrm{F}}. \tag{16}$$

The normalized transmission cross-sections were then obtained by normalization with the respective nanoaperture area

$$\hat{\sigma}_{\mathrm{t}} = \frac{\sigma_{\mathrm{t}}}{\pi \cdot r^2} \tag{17}$$

Section 6: Fluorescence enhancement analysis

The time-tagged time-resolved (TTTR) data containing the photon arrival times during the $60\ \mathrm{s}$ measurements were converted to fluorescence time traces by counting the number of photons within a $100\ \mathrm{ms}$ time bin. Each of these traces was measured on a fluorescent molecule in the case of the confocal reference measurements or on a single nanostructure. Figure S6 (a) shows the steps involved to obtain the count rate difference upon bleaching or blinking of a single molecule.

First, the fluorescence time traces were baseline corrected [3] to remove the influence of the continuous bleaching of background fluorescence. The discrete count rate differences between two consecutive data points δC were calculated to find unexpectedly strong signal changes through an outlier detection algorithm with outliers being defined as all values outside of the threshold interval $(v_{\mathrm{low}}, v_{\mathrm{up}})$. The thresholds were calculated such that for $\alpha = 95\%$ of the fluorescence time traces with n data points all values would be within the interval assuming a normal distribution of δC.Thus, the threshold values were computed through the normal percent point function (PPF)

$$v_{\mathrm{low}} = \mathrm{PPF}(P_1^{\mathrm{out}}/2), \tag{18}$$

$$v_{\mathrm{up}} = \mathrm{PPF}(1 - P_1^{\mathrm{out}}/2), \tag{19}$$

with P_1^{out} being the probability of a single time trace to contain at least one outlier

$$P_1^{\mathrm{out}} = 1 - \alpha^{1/n}. \tag{20}$$

For each of the detected outliers an analysis window of $t_{\mathrm{win}} = 1\ s$ was defined around the detected event in the baseline corrected time trace C. If multiple outliers were detected

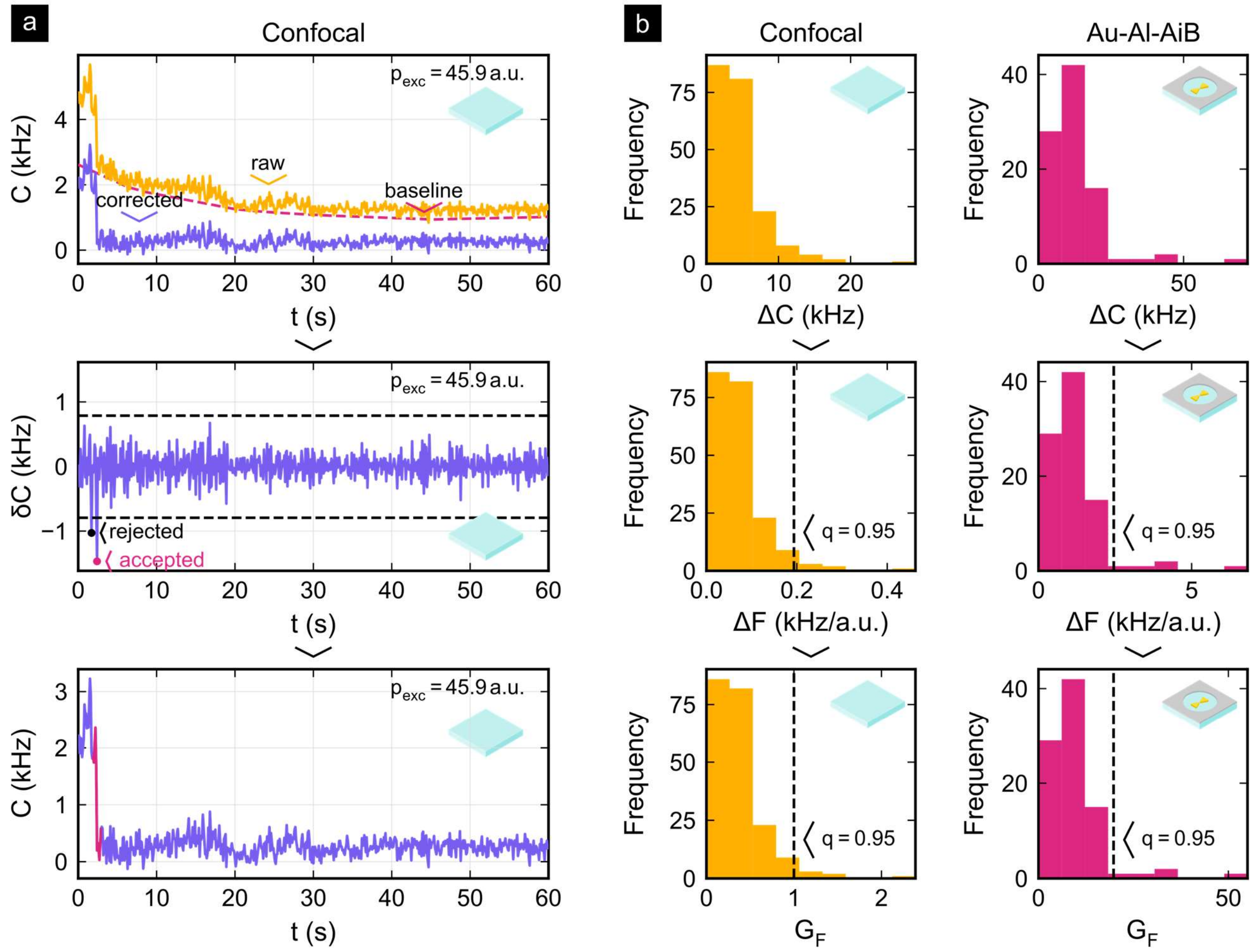

Figure S6. Detailed description of the blinking and bleaching detection. (a) The raw time traces were computed from the time-tagged time-resolved (TTTR) data with a binning time of $100\ \mathrm{ms}$ and then baseline corrected [3]. Outliers were detected in the discrete count rate differences δC through a threshold assuming a normal distribution. If multiple outliers were detected within the same analysis time window t_{win}, all points except the point with the highest absolute value were rejected. The step heights ΔC were computed by the absolute difference of the $q = 0.1$ and $q = 0.9$ quantiles of the baseline corrected trace C within $t_{\mathrm{win}}/2$ before and after the detected outlier points. (b) Histograms for ΔC were obtained by repeating this procedure for all recorded time traces. The fluorescence signal ΔF of a single molecule was computed by dividing ΔC by the excitation power p_{exc}. Dividing all fluorescence signals ΔF by the $q = 0.95$ quantile of the measured confocal fluorescence strengths yielded the fluorescence enhancements G_{F}.

within the same analysis window, all outliers besides the one with the largest absolute value were rejected to ensure the uniqueness of each event.

The step height ΔC of the bleaching or blinking event was calculated as the difference of the $q = 0.1$ and $q = 0.9$ quantiles of C within each analysis window. To account for the different excitation powers p_{exc}, the detected single molecule fluorescence signals were derived with

$$\Delta F = \frac{\Delta C}{p_{\mathrm{exc}}} \tag{21}$$

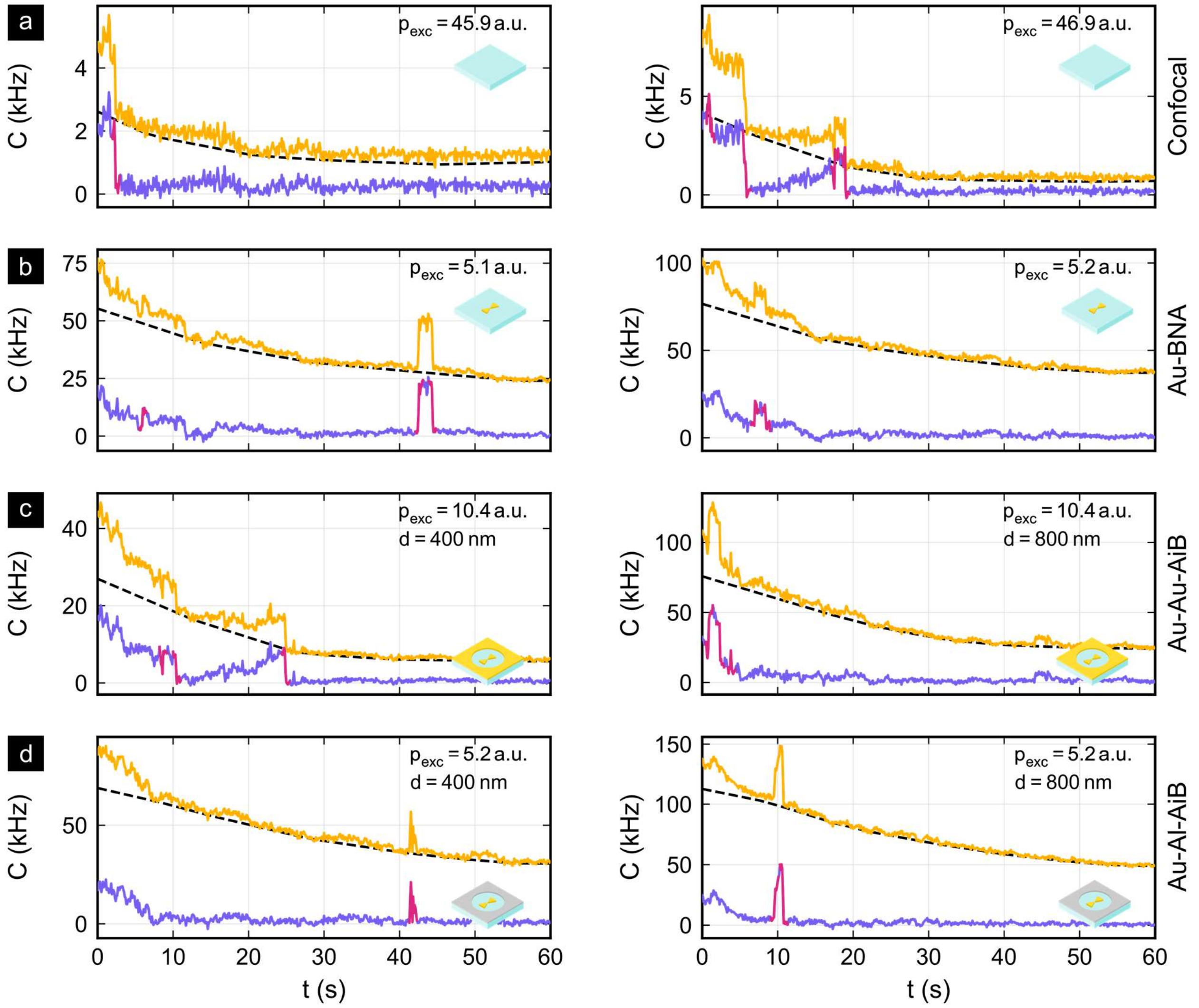

Figure S7. Exemplary fluorescence time traces for the different platforms. The raw time traces computed from the time-tagged time-resolved (TTTR) data are shown in yellow above the dashed black baseline. The purple baseline corrected fluorescence time traces have the detected blinking and bleaching steps overlaid in red. (a) shows two time traces for the confocal measurements at $10\ \mathrm{nM}$ dye concentration. In (b) two traces at two different BNA sites are displayed at $500\ \mathrm{nM}$ dye concentration. (c) and (d) show exemplary traces for the Au-Au- (c) and Au-Al-AiBs (d) for $d = 400\ \mathrm{nm}$ (left) and $d = 800\ \mathrm{nm}$ (right) both at $2000\ \mathrm{nM}$ dye concentration. The excitation powers for each trace are indicated on the top right.

from the step heights ΔC, yielding the fluorescence enhancement

$$G_F = \frac{\Delta F}{\Delta F_{\mathrm{C}}^{95}} \tag{22}$$

by dividing all fluorescence signals by the $q = 0.95$ quantile of the confocal fluorescence signal distribution $\Delta F_{\mathrm{C}}^{95}$.

Section 7: Exemplary fluorescence time traces and decay curves

Figure S7 displays additional exemplary fluorescence time traces that were used for the fluorescence enhancement analysis. The respective excitation power is displayed in

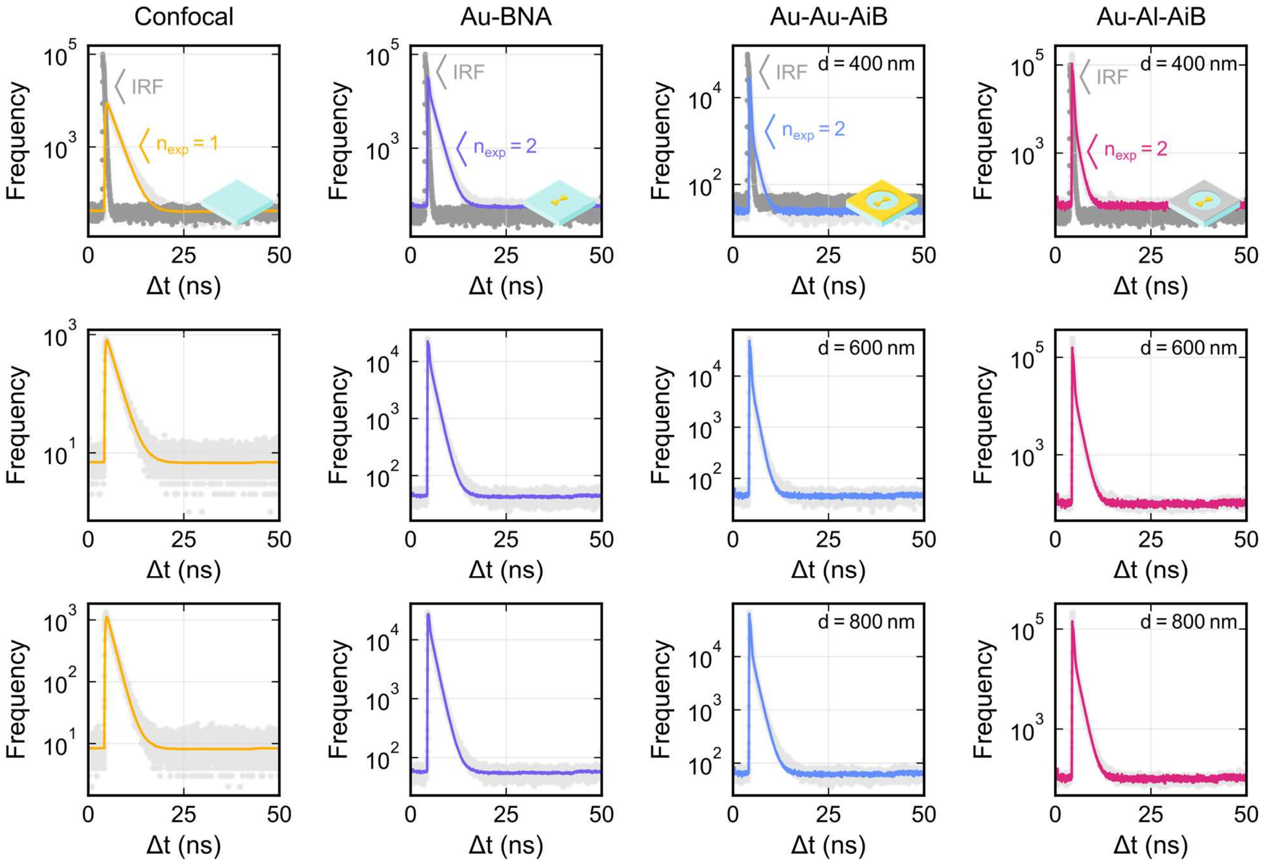

Figure S8. Exemplary fluorescence decay curves for the four different setups. Three fluorescence decay curve fits are shown for the confocal (yellow), Au-BNA (purple), Au-Au-AiB (blue), and Au-Al-AiB (red) setup. The experimental data is underlaid in light gray and the instrument response function (IRF, $\mathrm{FWHM} = 33\ \mathrm{ps}$) in dark gray in the top row. For the confocal curves a monoexponential decay was assumed whereas all other decays were fitted with a biexponential decay. For both AiB platforms the three decay curves are displayed for three different aperture radii indicated on the top right of each plot.

arbitrary units on the top right of each time trace and is linear to the actual excitation power as can be seen in Figure S9 (a). Higher powers were used for the confocal reference measurements to be able to clearly distinguish the blinking and bleaching of single molecules. For all time traces, the raw signal after applying the $100\ \mathrm{ms}$ time binning is shown in yellow. The estimated baseline [3] and corrected time traces are shown in black and purple, respectively. Detected blinking and bleaching events are highlighted in red. The different excitation powers and dye concentrations need to be considered when comparing the count rates at the start of each trace when no dyes are bleached yet. This gives an idea about the reduced background provided by the AiB platforms and especially the hybrid Au-Al-AiBs as well as the influence of the aperture diameter on the strength of the fluorescence background.

Exemplary decay curves (light gray) are displayed in Figure S8 together with the corresponding curve fits (colored) and the instrument response function (IRF, dark gray) that has a full-width half-maximum (FWHM) of $33\ \mathrm{ps}$. As pointed out in the main text the

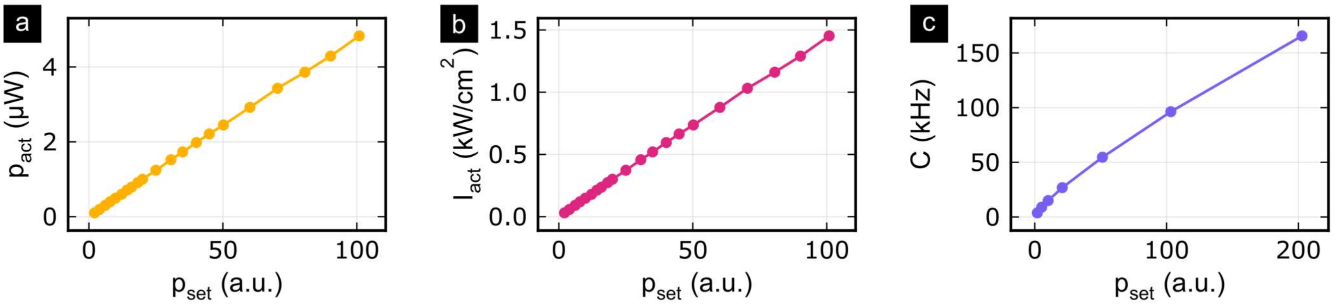

Figure S9. (a) shows the actual average power p_{act} before entering the objective in dependence of the set power $p_{\mathrm{set}} = p_{\mathrm{exc}}$ and (b) the average intensity I_{act} that was computed from the actual power for a diffraction limited beam with $\lambda_{\mathrm{exc}} = 640\ \mathrm{nm}$ and $\mathrm{NA} = 1.2$. The detected count rate in dependence of the excitation power is illustrated in (c). The count rate was measured confocally and defined as the average fluorescence count rate in a $30\ \mathrm{s}$ time trace of $50\ \mathrm{nM}$ Alexa Fluor™ 647 dissolved in Milli-Q® water.

data were fitted using a multiexponential decay model convoluted with the IRF. For the confocal reference curves, a single exponential decay was found to describe the data well, whereas in the case of the nanostructured samples a second component was required. The two components were attributed to strongly excited dyes in the BNA gap region (fast component) and dyes weakly interacting with the nanoantenna or nanoaperture (slow component). It can be seen that the fast component is much more pronounced in the case of the AiBs as compared to the BNAs.

Section 8: Excitation power characterization

Figure S9 (a) shows how the actual average power p_{act} relates to the set excitation power p_{set} in arbitrary units. The power was measured with a powermeter in the back focal plane of the objective. The set and actual powers are linearly dependent which is why the fluorescence enhancement analysis was done based on the set power $p_{\mathrm{set}} = p_{\mathrm{exc}}$ in arbitrary units. The intensities in (b) were calculated using the diameter of the Airy disk

$$d_{\mathrm{Airy}} = 1.22 \cdot \frac{\lambda_{\mathrm{exc}}}{\mathrm{NA}} \tag{23}$$

with $\lambda_{\mathrm{exc}} = 640\ \mathrm{nm}$ and $\mathrm{NA} = 1.2$ and the resulting area

$$A_{\mathrm{Airy}} = \pi \cdot \left(\frac{d_{\mathrm{Airy}}}{2}\right)^2 \tag{24}$$

as

$$I_{\mathrm{act}} = \frac{P_{\mathrm{act}}}{A_{\mathrm{Airy}}} \tag{25}$$

Even excitation powers of up to $100\ \mathrm{a.u.}$ correspond to intensities of only around $1.5\,\frac{\mathrm{kW}}{\mathrm{cm}^2}$. Despite the rather low excitation intensities, Figure S9 (c) shows a small nonlinear

dependence of the fluorescence count rate on the excitation power when measuring the average count rate C of a $30\ s$ time trace with $50\ \mathrm{nM}$ Alexa Fluor™ 647 dissolved in Milli-Q® water. Due to the relatively low intensities used we attribute this mainly to dark state saturation that does not affect the single molecule bleaching analysis.

References

(1) Novotny, L.; Hecht, B. *Principles of Nano-Optics*; Cambridge University Press: Cambridge, 2006. https://doi.org/10.1017/CBO9780511813535.

(2) Payne, L. M.; Langbein, W.; Borri, P. Polarization-Resolved Extinction and Scattering Cross-Sections of Individual Gold Nanoparticles Measured by Wide-Field Microscopy on a Large Ensemble. *Appl. Phys. Lett.* **2013**, *102* (13), 131107. https://doi.org/10.1063/1.4800564.

(3) Zhang, Z. M.; Chen, S.; Liang, Y. Z. Baseline Correction Using Adaptive Iteratively Reweighted Penalized Least Squares. *Analyst* **2010**, *135* (5), 1138–1146. https://doi.org/10.1039/b922045c.
